# Development and validation of a deep learning algorithm for improving Gleason scoring of prostate cancer

**DOI:** 10.1038/s41746-019-0112-2

**Published:** 2019-06-07

**Authors:** Kunal Nagpal, Davis Foote, Yun Liu, Po-Hsuan Cameron Chen, Ellery Wulczyn, Fraser Tan, Niels Olson, Jenny L. Smith, Arash Mohtashamian, James H. Wren, Greg S. Corrado, Robert MacDonald, Lily H. Peng, Mahul B. Amin, Andrew J. Evans, Ankur R. Sangoi, Craig H. Mermel, Jason D. Hipp, Martin C. Stumpe

**Affiliations:** 1 Google AI Healthcare, Google, Mountain View, CA USA; 2 Laboratory Department, Naval Medical Center San Diego, San Diego, CA USA; 3 Henry M. Jackson Foundation, Bethesda, MD USA; 4 Department of Pathology and Laboratory Medicine, University of Tennessee Health Science Center, Memphis, TN USA; 5 Department of Pathology, Laboratory Medicine and Pathology, University Health Network and University of Toronto, Toronto, ON Canada; 6 Department of Pathology, El Camino Hospital, Mountain View, CA USA; 7 Present Address: AI and Data Science, Tempus Labs Inc, Chicago, United States

**Keywords:** Prostate cancer, Prostate cancer

## Abstract

For prostate cancer patients, the Gleason score is one of the most important prognostic factors, potentially determining treatment independent of the stage. However, Gleason scoring is based on subjective microscopic examination of tumor morphology and suffers from poor reproducibility. Here we present a deep learning system (DLS) for Gleason scoring whole-slide images of prostatectomies. Our system was developed using 112 million pathologist-annotated image patches from 1226 slides, and evaluated on an independent validation dataset of 331 slides. Compared to a reference standard provided by genitourinary pathology experts, the mean accuracy among 29 general pathologists was 0.61 on the validation set. The DLS achieved a significantly higher diagnostic accuracy of 0.70 (*p* = 0.002) and trended towards better patient risk stratification in correlations to clinical follow-up data. Our approach could improve the accuracy of Gleason scoring and subsequent therapy decisions, particularly where specialist expertise is unavailable. The DLS also goes beyond the current Gleason system to more finely characterize and quantitate tumor morphology, providing opportunities for refinement of the Gleason system itself.

## Introduction

Adenocarcinoma of the prostate is the second most common cancer diagnosed in men, with approximately one in nine men diagnosed in their lifetime.^1^ For prostate cancer patients, subjective microscopic tissue examination remains the gold standard for diagnosis. Here, the Gleason score and tumor stage have remained the most powerful predictors of prognosis in virtually every large prostate cancer outcome study.^2^ The Gleason system was initially developed in 1966 and stratifies prostate malignancies by tumor architectural patterns. The system has since been revised significantly^3,4^ in an attempt to better reflect tumor biology. Importantly, the Gleason score (and its associated Gleason Grade Group^2^) is central to risk stratification and the National Comprehensive Cancer Network guidelines,^5^ which are widely used clinically to guide standardized patient management decisions. Despite its indisputable role in prognostication and patient management, Gleason scoring by pathologists is a subjective exercise and suffers from suboptimal interobserver and intraobserver variability, with reported Gleason score discordance ranging from 30% to 53%.^6–14^

A potential approach to increasing the consistency and accuracy of Gleason grading lies in the field of artificial intelligence, where recent advances using deep learning have been applied productively to imaging diagnostic tasks across dermatology,^15,16^ ophthalmology,^17–20^ radiology,^21–23^ and histopathology.^24–29^ Similarly, prior computational approaches have tackled Gleason grading using feature-engineering approaches,^30–32^ while more recent advances have applied deep learning to prostate cancer histopathology. These applications include binary classification on clinical specimens,^26,33^ and Gleason grading of tissue subsections^34,35^ or microarrays,^27,36^ which comprise carefully selected sub-regions of tumor specimens used for research purposes, outside of routine clinical workflow. This study complements prior studies by applying deep learning to conduct Gleason grading on entire clinical specimens, and also importantly uses an independent reference standard to compare algorithm accuracy to that of board-certified pathologists.

Expertise and consistency in Gleason scoring have been shown to significantly improve its prognostic utility.^9,37^ We thus reasoned that the availability of an accurate Gleason scoring tool for the whole-slide sections used in clinical workflows could help address the problem of grading variability, improve prognostication, and optimize patient management. To this end, we developed a deep learning system (DLS) to perform Gleason scoring and quantitation on prostatectomy specimens. The DLS accuracy is compared against a cohort of pathologists, where the reference standard was defined by genitourinary specialist pathologists. We further compared the risk stratification provided by our DLS, the cohort of pathologists, and our specialist-defined reference standard in predicting disease progression. Lastly, we also explored the potential of artificial intelligence to provide more fine-grained measures of tumor grading and the resulting potential to provide more precise prognostication.

## Results

### Overview of the deep learning system and data acquisition

**Fig. 1:**
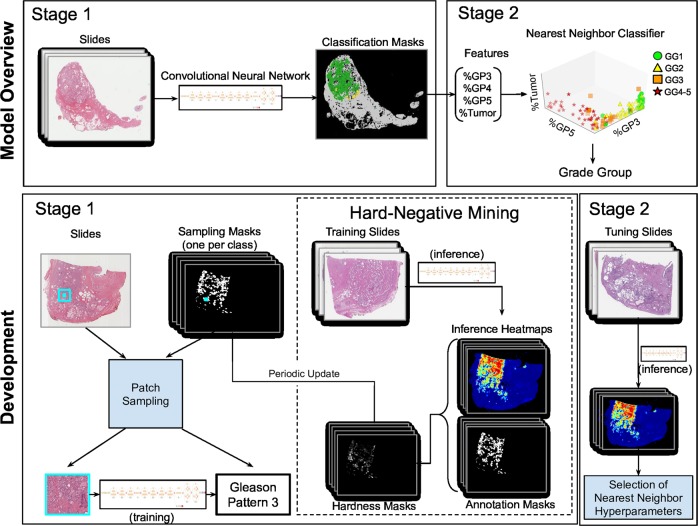
Illustration of the development and usage of the two-stage deep learning system (DLS). Developing the DLS involves training two machine learning models. Stage 1 is an ensembled deep convolutional neural network (CNN) that classifies every region in the slide as non-tumor or its Gleason pattern (GP). Training the stage 1 CNN involves first collecting pathologists’ annotations (Annotation Masks) of whole-slide images at the region level, and then generating “sampling masks” indicating the locations of each of the four classes (non-tumor, GP3, GP4, and GP5) for each slide. Over the course of millions of training iterations, sampled image patches and associated labels are used to train the constituent CNNs in the ensembled stage 1 CNN model. During the training process, we performed hard-negative mining by periodically applying each individual partially trained model to the entire training corpus of whole-slide images. Comparison of these intermediate inference results to the original annotations highlights the most difficult image patches, and we focus training on these patches. Stage 2 involves first collecting pathologists’ labels of the Gleason Grade Group (GG) for each slide. Next, the predictions of the stage 1 model are calibrated and converted to four features that indicate the amount of tumor and each GP in the slide. k-nearest-neighbor (kNN) classifiers are then trained to predict the GG (1, 2, 3, or 4–5), or whether the GG is above specific thresholds (GG ≥ 2, GG ≥ 3, or GG ≥ 4). For more details, please refer to the “Deep Learning System” section in the Supplement

Our approach is a two-stage deep learning system (DLS): first a deep convolutional neural network-based regional Gleason pattern (GP) classification followed by a k-nearest-neighbor-based whole-slide Gleason Grade Group classification (Fig. 1). The first stage was trained using image patches extracted from the slide and the corresponding label derived from pathologist-labeled pixel-level annotations (Fig. 1). In total, we collected and used 112 million image patches derived from 912 slides (approximately 115,000 mm^2^ of tissue), which required approximately 900 pathologist hours to annotate and is roughly 4× larger in annotated tissue area than the training slides in the widely used Camelyon16 dataset.^24^ The second stage was trained using 1159 slide-level classifications provided by pathologists.

The DLS was evaluated on an independent validation dataset collected from three sources, consisting of 331 slides from 331 patients (Table 1). At least three pathologists provided initial reviews for each slide. A genitourinary specialist pathologist subsequently reviewed each slide along with the initial pathologists’ comments to provide a final grade for use as the reference standard (Methods).

**Table 1:** Number and breakdown of slides in the validation dataset

	Source or diagnosis	TCGA	Tertiary teaching hospital	Medical laboratory	Total
Number of patients	Patients with available prostatectomy specimens	219	157	4	380
	Excluded due to non-gradable prostate cancer variants	22	5	0	27
	Excluded due to extensive image artifacts or poor staining	2	0	0	2
	Specialist unable to provide confident diagnosis	12	8	0	20
Slide-level Gleason Grade Group	Patients in study (1 slide per patient)	183	144	4	331 (100%)
	Grade Group 1	10	67	0	77 (23%)
	Grade Group 2	77	57	0	134 (40%)
	Grade Group 3	46	14	2	62 (19%)
	Grade Group 4–5	50	6	2	58 (18%)
	Grade Group 4	10	2	0	12 (4%)
	Grade Group 5	40	4	2	46 (14%)
Region-level Gleason pattern annotations	Number of slides	62	14	3	79
	Non-tumor (patches)	18,022,643	10,879,735	2,152,853	31,055,231
	Gleason pattern 3 (patches)	2,445,437	343,685	2,016	2,791,138
	Gleason pattern 4 (patches)	4,288,977	8,280	106,227	4,403,484
	Gleason pattern 5 (patches)	1,797,331	326	129,059	1,926,716

The validation set contains prostatectomy cases from three sources. A representative slide was selected from each patient’s case. The reference standard for the Gleason scores in the validation set was established by an initial review by at least three pathologists from a cohort of 19 and then adjudication by one of three genitourinary specialists. The low prevalence of Grade Groups 4 and 5 in our dataset prompted us to merge these two groups for more reliable statistical comparisons

### Comparison of DLS to pathologists on whole-slide Gleason scoring

**Fig. 2:**
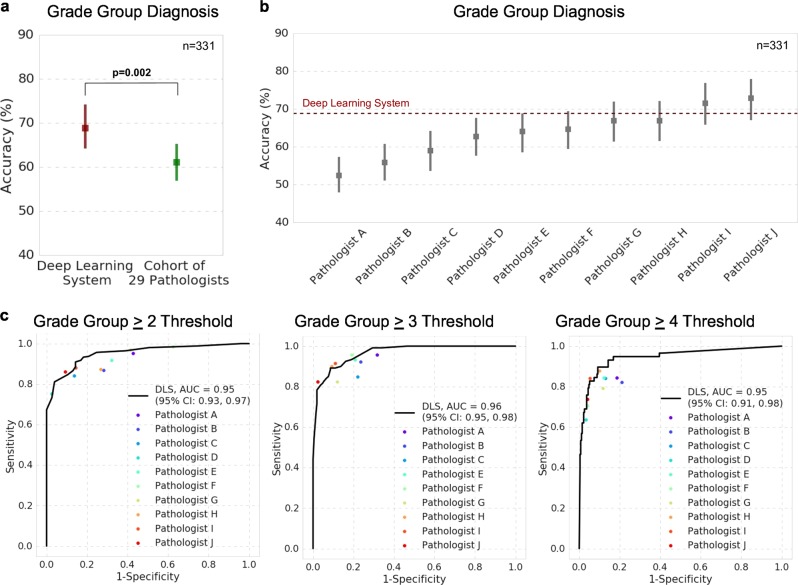
Comparison of prostate cancer Gleason scoring performance of the deep learning system (DLS) with pathologists. **a** Accuracy of the DLS (in red) compared with the mean accuracy among a cohort-of-29 pathologists (in green). Accuracy is defined as exact agreement with the reference standard, which is provided by genitourinary specialists (see Methods). Error bars indicate 95% confidence intervals, and *p*-value is the result of a two-sided permutation test (see “Statistical Analysis” section in the manuscript and the Supplement). **b** Accuracy of the DLS compared to 10 individual pathologists (among the cohort of 29, indicated by pathologists A–J) who reviewed all of the slides in the validation set. See eTable 4 in the Supplement for more details. **c** The receiver operating characteristic curves compare the sensitivity and specificity of the DLS with individual pathologists and the cohort-of-29 pathologists for binary classification of whether the Gleason Grade Group (GG) is above the thresholds of GG ≥ 2, GG ≥ 3, and GG ≥ 4. Area under the receiver operating characteristic curves and associated 95% confidence intervals for the DLS are provided in the legend. Higher and to the left indicates better performance

Independent of establishing the reference standard, we collected additional pathologist reviews on the validation dataset to compare with the DLS’s performance. The mean accuracy among the 29 pathologists in classifying each slide’s Gleason Grade Group was 0.61 (95% confidence interval (CI): 0.56–0.66). The DLS achieved an accuracy of 0.70 (95% CI 0.65–0.75), higher than the cohort of 29 (*p* = 0.002; Fig. 2a). A subgroup of 10 pathologists in this cohort reviewed the entire validation dataset, with individual accuracies ranged from 0.53 to 0.73 (mean: 0.64). The DLS was more accurate than 8 of these 10 pathologists (Fig. 2b; Supplementary Table 4). The remaining 19 pathologists reviewed overlapping subsets of the validation set (see Methods), achieving individual accuracies ranging from 0.31 to 0.74 (mean: 0.60). Additional analyses are presented in Supplementary Tables 5 and 6 and Supplementary Fig. 1.

We additionally looked at three Grade Group (GG) decision thresholds: GG ≥ 2, GG ≥ 3, and GG ≥ 4. The DLS achieved areas under the receiver operating characteristic curves (AUCs) of 0.95–0.96 at each of these thresholds (Fig. 2c). The largest difference occurred at the GG ≥ 4 threshold, where the DLS demonstrated both a higher sensitivity and specificity than 9 out of 10 individual pathologists.

### Comparison of DLS to pathologists on Gleason pattern quantitation

**Fig. 3:**
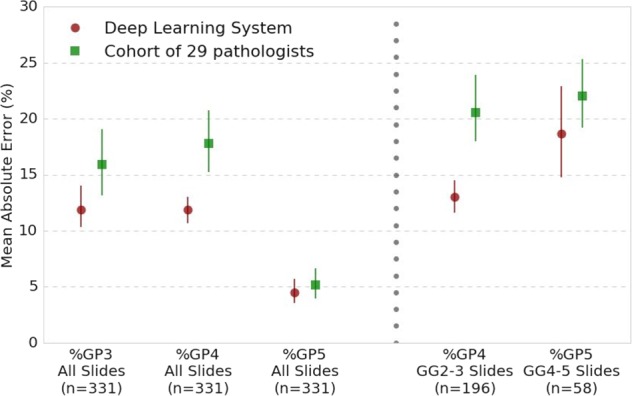
Comparison of the deep learning system (DLS) with pathologists for Gleason Pattern (GP) quantitation. Each dot indicates the mean average error (lower is better) for Gleason pattern quantitation, with error bars show the 95% confidence intervals. Left: overall Gleason pattern quantification results among all slides. Right: subgroup analysis where Gleason pattern quantification is of particular importance: Grade Group 2–3 slides where percent of Gleason pattern 4 can change the overall Grade Group, and Grade Group 4–5 slides where percent of Gleason pattern 5 reporting is recommended by the College of American Pathologists

In addition to the Grade Group, more granular reporting of the relative amounts of Gleason patterns is recommended by the International Society of Urological Pathology (ISUP), College of American Pathologists (CAP), World Health Organization (WHO), and recent publications.^38–41^ As such, we also compared the DLS’s accuracy in Gleason pattern quantitation to that of pathologists. Relative to the genitourinary pathologist reference standard, the DLS had a 4–6% lower mean absolute error (MAE) than the average pathologist for quantitation of patterns 3 and 4 (Fig. 3). In subgroup analysis, for slides in Grade Groups 2 and 3 (where the amount of pattern 4 can change the overall Grade Group), the DLS again achieved better quantitation (8% lower MAE). The trend for Grade Groups 4 and 5 (where quantitation of pattern 5 is significant) was similar. More details are available in Supplementary Tables 7 and 8.

### Insights from DLS region-level classifications

**Fig. 4:**
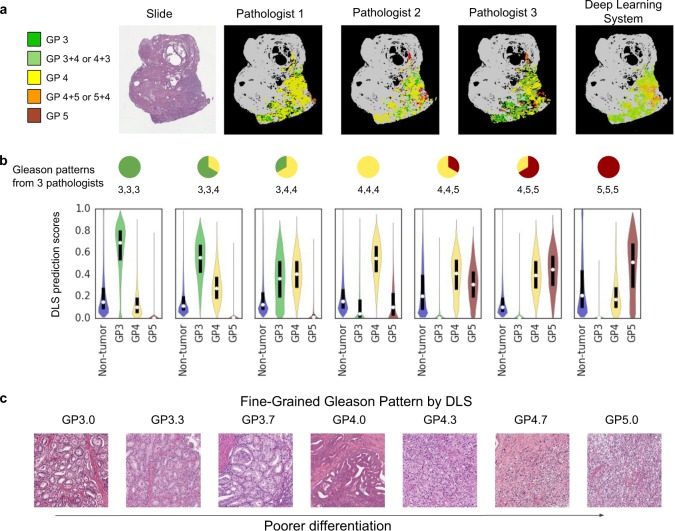
Assessing the region-level classification of the DLS. **a** Three pathologists annotated this slide with general concordance on the localization of tumor areas, but poor agreement on the associated Gleason patterns: a “pure” grade like Gleason pattern 3, 4, or 5, or a mixed grade comprising features of more than one pure pattern. The DLS assigned each image patch to a fine-grained Gleason pattern, as illustrated by the colors interpolating between Gleason patterns 3 (green), 4 (yellow), and 5 (red). See the “Fine-grained Gleason Pattern” section in the Supplement. **b** Quantification of the observations from panel **a** across 79 slides (41 million annotated image patches) for which three pathologists exhaustively categorized every slide. The violin plots indicate DLS prediction-likelihood distributions. The white dots and black bars identify medians and interquartile ranges, respectively. The predicted likelihood of each Gleason pattern by the DLS changes smoothly with the pathologists’ classification distribution. See Supplementary Fig. 2 for a similar analysis on images with mixed-grade labels. **c** The continuum of Gleason patterns learned by the DLS reveals finer categorization of the well-to-poorly differentiated spectrum (see “Fine-grained Gleason Pattern” section in the Supplement). Each displayed image region is the region closest (of millions in our validation dataset) to its labeled quantitative Gleason pattern. Columns 1, 4, and 7 represent regions for which the highest confidence predictions are Gleason patterns 3, 4, and 5, respectively. The columns in between represent quantitative Gleason patterns between these defined categories. See Supplementary Fig. 3 for additional examples

Furthermore, we evaluated the DLS’s ability to classify tissue regions within each slide. We collected exhaustive region-level annotations for 79 slides, performed by three pathologists per slide, and compared the predictions of the DLS to these annotations (see Fig. 4a for an example). We first characterized the DLS’s predictions by examining regions where the pathologists were concordant. For regions where all three pathologists agree on the same region classification (one of: non-tumor, Gleason pattern 3, 4, or 5), the DLS concurs 97% of the time. For the subset of these regions classified as a Gleason pattern, the DLS favors the same Gleason pattern as the pathologists 88% of the time (see Supplementary Results for an analysis of DLS errors).

Next, we characterized the DLS’s prediction for regions where the pathologists were discordant by plotting the confidence score of the DLS for each category as a function of inter-pathologist agreement (Fig. 4b and Supplementary Fig. 2). For tissue regions where pathologists are concordant on Gleason pattern 3, discordant between 3 and 4, or concordant on Gleason pattern 4, the DLS’ prediction scores change smoothly with the pathologists’ classification distribution. The same trend is seen as we move from Gleason pattern 4 to 5. We further used the DLS’s prediction scores directly to classify regions as *fine-grained Gleason patterns* (e.g. Gleason patterns 3.3 or 3.7). We found that by doing so, that DLS was able to represent a more gradual transition from well-to-poor differentiation than allowed by the canonical coarse Gleason pattern buckets (Fig. 4c; Supplementary Fig. 3).

### Measuring effectiveness of Gleason scoring in risk stratification for disease progression

**Fig. 5:**
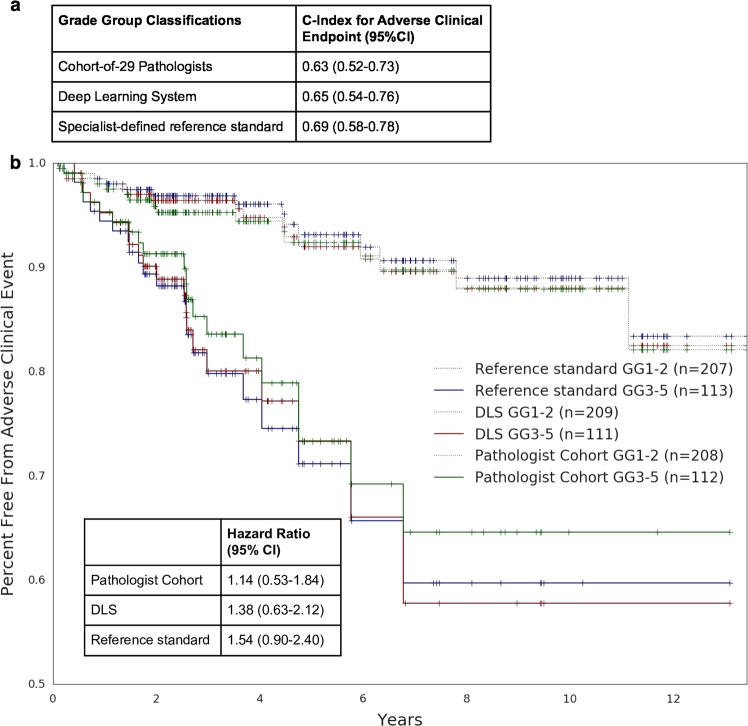
Comparison of risk stratification between pathologists, deep learning system, and the specialist-defined reference standard. **a** Concordance index provided by each entity’s Grade Group (GG) classification (GGs 1, 2, 3, 4–5) in stratifying adverse clinical endpoints of disease progression or biochemical recurrence (BCR) (see “Clinical Follow-up Data” in Methods). Ninety-five percent confidence intervals were obtained by bootstrapping. For the cohort-of-29 pathologists, the median c-index is reported (see “Statistical Analysis” in Supplementary Methods). **b** Kaplan–Meier curves using a binary threshold (GG ≥ 3) for risk stratification. Dotted lines correspond to the lower risk group (GG1-2) and solid lines correspond to the higher risk group (GG3-5). A larger separation between the risk groups indicates better risk stratification. Tick marks indicate censorship events. For the cohort-of-29 pathologists, analyses of sampled Grade Group classifications that produced a median hazard ratio are plotted here (see “Statistical Analysis” in Supplementary Methods)

Lastly, we compared the ability of the DLS, the cohort of pathologists, and genitourinary specialist pathologists (who comprised the reference standard) to risk stratify patients for biochemical recurrence or disease progression (see Methods). In this analysis, we measured prognostic performance using the *c-index*, which is an extension of AUC that handles censored data in survival analysis. On the validation set, the DLS-predicted Gleason Grade Group achieved a *c*-index of 0.65. The pathologist-provided Grade Groups yielded a median *c*-index of 0.63 (see Methods), while the genitourinary specialist pathologists achieved a *c*-index of 0.69. Kaplan–Meier and hazard ratio analyses using a binary GG ≥ 3 threshold, where hazard ratios for GG3 have previously been shown to be three-fold higher than GG2,^2^ to stratify patients into “high risk” and “low risk” categorizations showed the same trend (Fig. 5).

In addition to the risk stratification performance of Grade Groups, we also used Cox models^42^ to evaluate the prognostic ability of the underlying quantified Gleason patterns. The *c*-indices of these models were 0.697 for the DLS, 0.674 for the cohort of 29 pathologists, and 0.690 for the specialist-defined reference standard. As proof of concept that finer-grained Gleason patterns can improve risk stratification, we also evaluated Cox-regression models trained on a more granular representation of the tumor pattern composition. Adding “GP3.5” to the canonical Gleason patterns (thus summarizing the tumor composition as %GP3, %GP3.5, %GP4, and %GP5) raised the *c*-index to 0.704. Further adding %GP4.5 resulted in a *c*-index of 0.702 (Supplementary Table 10).

## Discussion

The present study shows that a DLS was more accurate than a cohort of 29 board-certified pathologists in Gleason scoring whole-slide images from prostatectomy patients. The pathologists in this study had a 66% Gleason score concordance (61% Gleason Grade Group concordance) with genitourinary specialist pathologists, which is at the high end of several reported inter-pathologist Gleason score concordances of 47–70%.^6–14^

Previous studies have highlighted the value of expertise in pathologic interpretation. Central histologic reviews provided by pathologists experienced in genitourinary pathology improved prognostication relative to reviews provided by the local institution. Encouragingly, the risk stratification performance (as measured by the *c*-index and hazard ratio) in this study followed the same trend.^9,37^ Due to the importance of genitourinary expertise in pathologic review, a second review has been recommended for high-risk patients after prostatectomy and for needle biopsies prior to prostatectomy.^8,9,43^ In routine pathologic workflows, DLS-predicted Gleason scores could be computed on-demand and serve as a decision support tool. Future research is necessary to evaluate the potential clinical impact of the use of these predicted Gleason scores for patient prognostication and associated therapy decisions.

We further explored the implications of the DLS on each step of Gleason scoring and their respective scoring variability. The first aspect of Gleason scoring is the region-level classification of Gleason patterns across each slide. In this step, two-dimensional histologic examination of the three-dimensional tissue structures creates inherent ambiguity. Substantial additional variability arises from applying discrete categorizations to glandular differentiation that lies on a continuous spectrum, such as the Gleason pattern 3/4 transition between small glands and poorly defined acinar structures or the Gleason pattern 4/5 transition between fused glands and nests or cords.^12,44,45^ Our data show that for regions where pathologists are discordant in Gleason pattern categorization, where the underlying histology is likely closer to the cusp between patterns, the DLS reflects this ambiguity in its prediction scores (Fig. 4b) and demonstrates the potential to assign finer-grained Gleason patterns (Fig. 4c). This finer-grained categorization provides opportunities to mitigate variability stemming from coarse categorization of a continuum, and opens avenues of research for more precise risk stratification (see Supplementary Table 10).

The next step in Gleason scoring after region-level categorization involves visual quantitation of the relative amounts of each Gleason pattern to determine the most prevalent patterns. Quantitation also allows for more granular prognostication. For example, prior studies have shown that prognosis of Grade Group 2–3 patients worsened for increases of percent Gleason pattern 4 as small as 5–10%.^41^ As such, reporting of the quantitation of Gleason patterns is recommended.^4,38,46^ However, visual quantitation is associated with inherent subjectivity.^47^ In this regard, the DLS bypasses the variability introduced by visual quantitation through direct quantitation of Gleason patterns from its underlying region categorizations. The DLS’s natural advantage in this regard and its more accurate quantitation than the cohort of pathologists (as measured by agreement with a specialist-adjudicated reference standard) suggest opportunity for more precise prognostication.

The above results complement previous works on the application of deep learning to prostate cancer histopathology. Campanella et al.^26^ demonstrated the use of deep learning in needle core biopsies to facilitate the detection of cancer foci. Arvaniti et al.^27^ applied deep learning to Gleason score tissue microarrays. This study complements prior work by applying deep learning to Gleason grading specimens that are more representative of a diversity of histologies and artifacts seen in routine clinical practice, and by directly comparing algorithmic performance with pathologists on a large multi-institutional dataset, with a rigorous reference standard defined by a team of board-certified pathologists and genitourinary specialist pathologists.

Another notable aspect of our work is the complexity and scale of the annotations required to develop our DLS. The complexity of Gleason grading has been discussed above; formalizing these interpretations as concrete annotations for training the DLS involved significant complexity, for example, “mixed” Gleason grades, artifacts, non-prostate tissue such as seminal vesicles, pre-malignant tissue, and uncommon variants. Please see Methods and Supplementary Methods for our detailed protocol. The size of this dataset was a key contributor to the accuracy of our DLS; training different models on titrated fractions of our dataset suggests that the DLS performance benefited greatly from the size of the dataset, and may yet improve with more or better quality data. Given the interobserver variability in Gleason grading, we also increased the accuracy of the pixel-level annotations in our tuning set by collecting triplicate annotations for each slide (see Methods and Supplementary Methods for details about the annotation and DLS training protocol).

In addition, our DLS stage-1 development process includes large scale, continuous “hard-negative mining” which aims to improve algorithm performance by running inference on the entire training dataset to isolate the hardest examples and further refine the algorithm using these examples. For histopathology applications on whole-slide imaging, this is a computationally expensive process, requiring inference over 112 million image patches in our training dataset. While previous works employing deep learning on histopathology images have employed hard-negative mining in an offline “batch-mode”,^24,48,49^ we observed that performance improves with the frequency of inference on the entire training dataset, resulting in the “quasi-online” hard-negative mining approach (>30,000 DLS stage-1 inferences per second) used here. We anticipate that the benefits of this continuous hard-negative mining approach may also be applicable to developing other histopathology deep learning algorithms.

This study has important limitations that would need to be addressed prior to implementation of associated tools in clinical practice. First, although clinical environments are currently still largely based on glass slide review, this study focuses on digital review. Next, in addition to conducting Gleason grading, pathologists are simultaneously carrying out several analyses, including staging and reviewing for unusual pathology. Though DLS grading for each slide only requires a few minutes, the ideal integration of this computation into the pathology workflow (such as overnight, post-scanning, or on-demand) merits future study. Additionally, clinical environments enable pathologists to review sections, stains, or order consults for challenging cases. To account for this aspect, pathologists were asked to indicate when they would prefer additional resources or consults to provide a more confident diagnosis. Corresponding sensitivity analysis excluding these cases is provided in Supplementary Table 9, showing qualitatively similar results.

Next, this study focuses on grading acinar prostatic adenocarcinoma (the vast majority of prostate cancer cases) in prostatectomy specimens, where the Grade Group informs postoperative treatment decisions rather than the decision to undergo the prostatectomy itself. As such, clinical outcomes after prostatectomy are less confounded by divergent treatment pathways than biopsies, supporting analyses of correlations with clinical follow-up data. In addition, prostatectomy specimens contain more tissue than biopsies, providing greater context during histological examination and improving the quality of the reference standard. However, important future work will generalize and validate the DLS for biopsies, other histologic variants, and other prognostic categorizations to aid clinical decisions throughout prostate cancer treatment pathways. Lastly, validation on larger clinically annotated datasets is required to evaluate the statistical significance of trends associated with prognostication demonstrated in this work.

In conclusion, we have developed a DLS that demonstrated greater accuracy than a cohort of 29 generalist pathologists in Gleason scoring prostatectomy whole-slide images. Additionally, the DLS provides more accurate quantitation of Gleason patterns, finer-grained discretization of the well-to-poor differentiation spectrum, and opportunities for better risk stratification. In doing so, our DLS demonstrates the potential to enhance the clinical utility of the Gleason system for better treatment decisions for patients with prostatic adenocarcinoma.

## Methods

### Acquisition of data

De-identified, digitized whole-slide images of hematoxylin-and-eosin (H&E)-stained formalin-fixed paraffin-embedded (FFPE) prostatectomy specimens were obtained from three sources: a public repository (The Cancer Genome Atlas, TCGA,^50^ *n* = 397 patients), a large tertiary teaching hospital in the US (Naval Medical Center San Diego, NMCSD, *n* = 361 patients), and an independent medical laboratory (Marin Medical Laboratories, *n* = 11 patients; Table 1; Supplementary Table 1). The study protocol was approved and informed consent was waived by the NMCSD Institutional Review Board (IRB), #NMCSD.2012.0091, because the data were de-identified and used for a retrospective study without impacting patient care. This IRB covered the use of anonymized cases independent of data source for the purposes of this study. For the TCGA, we used all available formalin-fixed paraffin-embedding (FFPE) surgical resection cases from the “PRAD” (prostate adenocarcinoma) study.

From TCGA we included all FFPE prostatectomy cases, the slides for which were scanned using a mix of scanners, including both Aperio and Hamamatsu scanners, and a mix of resolutions: ≈0.25 µm/pixel (“×40 magnification”) and ≈0.5 µm/pixel (“×20 magnification”). From the hospital we included all prostatectomy cases where FFPE tissue blocks or slides were available based on a review of de-identified pathology notes. From the independent laboratory we obtained additional cases based on pathology reports to improve the representation of Gleason Grade Groups 4–5 in our study cohort (Table 1). From these sources, slides were obtained for cases within the 10-year Clinical Laboratory Improvement Amendments (CLIA) archival requirement, and tissue blocks for deaccessioned cases. Blocks were cut to produce sections of five-micron thickness and stained by CLIA-certified commercial laboratories (San Diego Pathology, San Diego, CA and Marin Medical Laboratories, Greenbrae, CA). Slides were digitized using a Leica Aperio AT2 scanner at a resolution of 0.25 µm/pixel.

Cases were randomly assigned to either the development (training/tuning) or independent validation datasets. For the 380 cases assigned to the validation dataset, pathologists identified one representative tumor-containing slide per case (see Grading section). Among these slides, 27 were excluded due to the presence of prostate cancer variants (Supplementary Table 2), 2 due to extensive artifacts or poor staining that hindered diagnosis, and 20 because of the inability of a genitourinary pathology specialist to confidently assign a diagnosis (Supplementary Table 3). The final validation dataset consisted of the remaining 331 slides (*n* = 183 from TCGA, *n* = 144 from the hospital, and *n* = 4 from the laboratory).

### Overview of pathologists’ annotations and reviews

A total of 35 pathologists reviewed slides for this study, all of whom completed residency in human anatomical pathology. Twenty-nine pathologists were US-board-certified (the “cohort of 29”) and another three had genitourinary specialization (one Canadian-board-certified and two US-board-certified). The remaining three pathologists were formerly board-certified or certified outside of North America, and provided annotations for the training and tuning datasets but not the validation dataset.

We collected slide-level reviews and region-level annotations from pathologists. Slide-level reviews categorize each slide into its Gleason Grade Group. Region-level annotations label specific tissue regions (such as specific Gleason patterns) within a slide. We describe the annotation protocol for the validation dataset here, and include additional details and the protocol for the training and tuning datasets in the “Grading” section and Supplementary Figure 5 in the Supplement.

### Collection of slide-level reference standard

The slide-level reference standard was used to validate the DLS’s and general pathologists’ performance. For each slide, the reference standard was provided by one genitourinary specialist pathologist. To improve accuracy, the specialist reviewing each slide also had access to initial Gleason pattern percentage estimates and free-text comments from prior reviews of at least three general pathologists. The specialist then determined the final GP percentages for tumor of each Gleason Pattern (GP): %GP3, %GP4, and %GP5 for use as the reference standard. We derived the slide-level Gleason score and corresponding Grade Group (1, 2, 3, or 4–5) based on the predominant and next-most-common Gleason patterns provided by the genitourinary specialist, avoiding variability introduced by inconsistent application of “tertiary replacement” (see “Grading” in the Supplement). All slides were reviewed in a manner consistent with ISUP 2014 and CAP guidelines with no time constraint.^4,38^

### Collection of slide-level reviews for pathologists’ performance

To evaluate general pathologists’ performance at Gleason scoring, we collected additional slide-level reviews for each slide, independent from those collected for determining the reference standard. These reviews came from a total of 29 pathologists. From this cohort, 10 pathologists provided reviews for every slide in the validation dataset. The remaining 19 pathologists reviewed overlapping subsets of the validation set (median: 53 slides, range: 41–64), collectively providing three reviews per slide.

These 29 pathologists represented varying experience levels (median years since anatomic pathology fellowship: 10, range: 1–37) and are distributed across 11 states in the US, coming from a combination of academic medical centers and independent pathology practices (see Supplementary Tables 4 and 5). Among 20 of these pathologists who responded to a follow-up survey about monthly prostate case volume, 35% of pathologists reported reviewing ≤10 cases, 45% reported reviewing 10–20, and 20% reported reviewing >20 cases monthly (see Supplementary Tables 4 and 5).

### Collection of region-level annotations

To compare region-level DLS predictions to pathologist interpretations, pathologists provided annotations of specific tissue regions within a slide, outlining individual glands or regions and providing an associated label (non-tumor, or GP3, 4, or 5). For these time-consuming region-level annotations, a subset of the validation dataset (79 of 331 slides) was selected based on slide-level Grade Group diversity. Each of these 79 slides was exhaustively annotated by three pathologists (≥95% tissue coverage; taking on average 3 h per pathologist per slide). Only regions for which all three pathologists provided a label were used for validation.

### Clinical follow-up data

To measure risk stratification performance, we used additional clinical follow-up data. For the TCGA subset of data, we used the progression-free interval as the clinical endpoint, as recommended by the authors of the TCGA Clinical Data Resource.^51^ For the hospital subset, biochemical recurrence, as defined by a postoperative prostate-specific antigen measurement of ≥0.4,^52^ was used as the clinical endpoint. Clinical endpoints were not available from the medical laboratory and for a small number of cases from TCGA and the hospital. Of the 331 validation slides, 320 had available clinical follow-up data.

### Deep learning system

The DLS consists of two stages (Fig. 1), which correspond to the region-level annotations and slide-level reviews: first a regional classification, and subsequent whole-slide Gleason Grade Group classification. The first stage segments each slide into small image patches and feeds each patch into a convolutional neural network that classifies each patch as one of four classes: non-tumor, or Gleason pattern 3, 4, or 5. When applied to the entire whole-slide image, this stage outputs a “heatmap” indicating the categorization of each patch in the tissue section. The second stage consists of a nearest-neighbor classifier that uses a summary of the heatmap output from the first stage to categorize the Grade Group of each slide. We briefly outline the DLS development procedure below, and provide additional details in the “Deep Learning System” section in the Supplement.

The first stage’s convolutional neural network is an InceptionV3 (ref. ^53^) network modified to be fully convolutional for inference computational efficiency as previously described.^54^ This network classifies each tissue region of roughly 32 × 32 µm by using input image patches of 911 × 911 µm centered on the region. The label for each region was derived from the pathologist-provided region-level annotations (see Supplementary Methods, “Grading” section). Ensembling and hard-negative mining were employed to further improve model performance (see Supplementary Methods, “Hard-negative Mining” section). Color normalization^55^ and alternate convolutional neural network architectures^56,57^ were included in experiments but showed no benefit.

In the second stage of the DLS, we first obtained a categorical prediction for each region by taking the class with the highest calibrated likelihood, where calibration weights were determined empirically using the tuning set. Next, for each slide, the number of regions predicted as each category was summarized and used for evaluation of (GP) quantitation (%GP3, %GP4, and %GP5). The three %GPs, together with the tumor involvement, were used as features (Fig. 1), similar to what a pathologist would need for Gleason scoring. Finally, we trained k-nearest-neighbor classifiers for several prediction tasks: four-class Grade Group (GG) classification (1, 2, 3, or 4–5), and each of three binary classifications of GG ≥ 2, GG ≥ 3, and GG ≥ 4. Support vector machines, random forest classifiers, and logistic regression were also included in experiments. The k-nearest-neighbor classifier was ultimately chosen for its high performance on the tuning set and its model simplicity (see Fig. 1).

### Statistical analysis

We assessed the DLS’s Gleason scoring performance relative to the reference standard for slide-level and region-level classifications. For slide-level Grade Group categorization, we compared the accuracy of the DLS to the mean of the 29 individual pathologist accuracies, where accuracy is the fraction of exact matches with the reference standard. This provided equal representation of each pathologist despite their differing number of reviews. We additionally measured performance using accuracy adjusted by a population-level Grade Group distribution^2^ and Cohen’s kappa.^58^ For the three binary classifications of slide-level Grade Group, we used the AUC. For quantitation of relative Gleason patterns in the tumors, we computed the MAE.

For clinical follow-up analysis, the concordance index was used to measure the overall effectiveness of Grade Group risk stratification with respect to an adverse clinical endpoint (disease progression or biochemical recurrence as described above). The hazard ratio and associated Kaplan–Meier curves were used to evaluate risk stratification at the binary classification of GG ≥ 3. For these risk stratification analyses, the cohort-of-29 pathologists Grade Group classifications were sampled to approximate equal representation of each pathologist (see “Statistical analysis” in the Supplement). Analysis on the sampled classifications that produced the median concordance and hazard ratios respectively among 999 sampling iterations is reported here.

Confidence intervals for all evaluation metrics were computed using a bootstrap approach (see “Statistical analysis” in the Supplement). All statistical tests were two-sided permutation tests. A *p*-value <0.05 was considered statistically significant. No adjustment for multiple comparisons was made. These analyses were performed in Python (v2.7.6), using the scikit-learn (v0.19.1) and lifelines (v0.12.0) libraries.

### Disclaimer

The views expressed in this article are those of the author(s) and do not necessarily reflect the official policy or position of the Department of the Navy, Department of Defense, nor the U.S. Government. A.M., N.O., J.L.S. and J.H.W. are military Service members. This work was prepared as part of their official duties. Title 17, U.S.C., §105 provides that copyright protection under this title is not available for any work of the U.S. Government. Title 17, U.S.C., §101 defines a U.S. Government work as a work prepared by a military Service member or employee of the U.S. Government as part of that person’s official duties.

### Reporting Summary

Further information on experimental design is available in the Nature Research Reporting Summary linked to this article.

## Supplementary information

Reporting Summary Checklist

Supplemental Materials

## Data Availability

The dataset from TCGA that was used in this study is available from the Genomic Data Commons portal (https://portal.gdc.cancer.gov/), which is based upon data generated by the TCGA Research Network (http://cancergenome.nih.gov/). The other datasets are not publicly available at this time due to restrictions in the data sharing agreements with the data sources. Ethics approval for the use of these de-identified slides in this study was granted by the Naval Medical Center San Diego Institutional Review Board (IRB).

## Supplementary information

**Supplementary information** accompanies the paper on the *npj Digital Medicine* website (10.1038/s41746-019-0112-2).

## Code availability

The deep learning framework used here (TensorFlow) is available at https://www.tensorflow.org/. The Python libraries used for computation and plotting of the performance metrics (SciPy, NumPy, Lifelines, and MatPlotLib) are available under https://www.scipy.org/, http://www.numpy.org/, https://github.com/CamDavidsonPilon/lifelines/, and https://matplotlib.org/, respectively.
