# Publisher Correction: Development and validation of a deep learning algorithm for improving Gleason scoring of prostate cancer

**DOI:** 10.1038/s41746-019-0196-8

**Published:** 2019-11-19

**Authors:** Kunal Nagpal, Davis Foote, Yun Liu, Po-Hsuan Cameron Chen, Ellery Wulczyn, Fraser Tan, Niels Olson, Jenny L. Smith, Arash Mohtashamian, James H. Wren, Greg S. Corrado, Robert MacDonald, Lily H. Peng, Mahul B. Amin, Andrew J. Evans, Ankur R. Sangoi, Craig H. Mermel, Jason D. Hipp, Martin C. Stumpe

**Affiliations:** 1 Google AI Healthcare, Google, Mountain View, CA USA; 2 Laboratory Department, Naval Medical Center San Diego, San Diego, CA USA; 3 Henry M. Jackson Foundation, Bethesda, MD USA; 4 Department of Pathology and Laboratory Medicine, University of Tennessee Health Science Center, Memphis, TN USA; 5 Department of Pathology, Laboratory Medicine and Pathology, University Health Network and University of Toronto, Toronto, ON Canada; 6 Department of Pathology, El Camino Hospital, Mountain View, CA USA; 7 Present Address: AI and Data Science, Tempus Labs Inc, Chicago, United States

**Keywords:** Prostate cancer, Prostate cancer

**Correction to:** *npj Digital Medicine* 10.1038/s41746-019-0112-2, published online 7 June 2019

The original version of the published Article omitted an affiliation for the last Author, Martin C. Stumpe. The last Author’s affiliation has been updated to include Google AI Healthcare, Google, Mountain View, CA, USA. This has been corrected in the HTML and PDF version of the Article.

