# Supplementary material for: Development and validation of a deep learning algorithm for improving Gleason scoring of prostate cancer
Source: NPJ Digit Med. 2019 Jun 7;2:48. doi: 10.1038/s41746-019-0112-2 (PMC6555810; doi:10.1038/s41746-019-0112-2)
Supplement: Supplementary file 1 — Reporting Summary Checklist [file 41746_2019_112_MOESM1_ESM.docx]

1

Corresponding author(s): Martin Stumpe (stumpem@gmail.com)


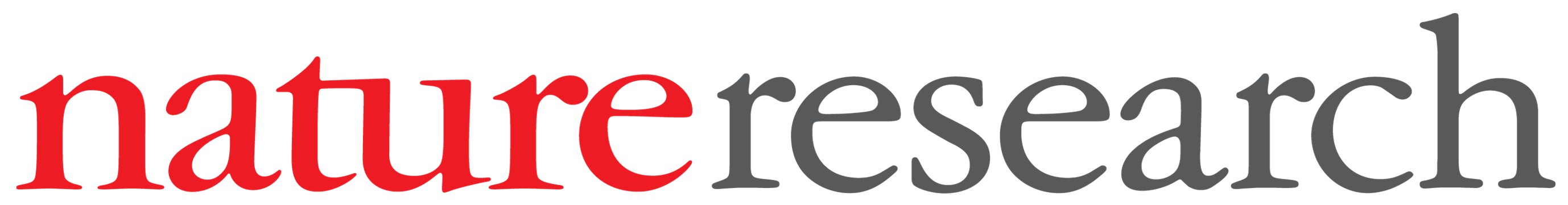


nature research | reporting summary

Initial submission Revised version Final submission

Reporting Summary

Nature Research wishes to improve the reproducibility of the work that we publish. This form provides structure for consistency and transparency in reporting. For further information on Nature Research policies, see Authors & Referees and the Editorial Policy Checklist.

Please do not complete any field with "not applicable" or n/a. Refer to the help text for what text to use if an item is not relevant to your study. For final submission: please carefully check your responses for accuracy; you will not be able to make changes later.

## Statistical parameters

When statistical analyses are reported, confirm that the following items are present in the relevant location (e.g. figure legend, table legend, main text, or Methods section).

n/a Confirmed


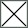

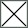

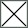


The exact sample size (*n*) for each experimental group/condition, given as a discrete number and unit of measurement

An indication of whether measurements were taken from distinct samples or whether the same sample was measured repeatedly The statistical test(s) used AND whether they are one- or two-sided

*Only common tests should be described solely by name; describe more complex techniques in the Methods section.*

A description of all covariates tested

A description of any assumptions or corrections, such as tests of normality and adjustment for multiple comparisons

A full description of the statistics including central tendency (e.g. means) or other basic estimates (e.g. regression coefficient) AND variation (e.g. standard deviation) or associated estimates of uncertainty (e.g. confidence intervals)

For null hypothesis testing, the test statistic (e.g. *F*, *t*, *r*) with confidence intervals, effect sizes, degrees of freedom and *P* value noted

*Give P values as exact values whenever suitable.*

For Bayesian analysis, information on the choice of priors and Markov chain Monte Carlo settings

For hierarchical and complex designs, identification of the appropriate level for tests and full reporting of outcomes Estimates of effect sizes (e.g. Cohen's *d*, Pearson's *r*), indicating how they were calculated

Clearly defined error bars

*State explicitly what error bars represent (e.g. SD, SE, CI)*

*Our web collection on statistics for biologists may be useful.*

## Software and code

Policy information about availability of computer code Data collection

Algorithms were developed in Python (v.2.7.6) using Tensorflow (v.1.11.0) available [at https://www.tensorflow.org/.](http://www.tensorflow.org/)

Data analysis

Analyses were performed in Python (v2.7.6), using the scikit-learn (v0.19.1) and lifelines (v0.12.0) libraries, available at https:// [www.scikit-learn.org](http://www.scikit-learn.org/) and https://github.com/CamDavidsonPilon/lifelines/ respectively.

For manuscripts utilizing custom algorithms or software that are central to the research but not yet described in published literature, software must be made available to editors/reviewers upon request. We strongly encourage code deposition in a community repository (e.g. GitHub). See the Nature Research guidelines for submitting code & software for further information.

## Data

Policy information about availability of data

*April 2018*

All manuscripts must include a data availability statement. This statement should provide the following information, where applicable:

- Accession codes, unique identifiers, or web links for publicly available datasets
- A list of figures that have associated raw data
- A description of any restrictions on data availability

The dataset from TCGA that was used in this study is available from the Genomic Data Commons portal (https://portal.gdc.cancer.gov/), which is based upon data

2

generated by the TCGA Research Networ[k (http://cancergenome.nih.gov/).](http://cancergenome.nih.gov/)) The other datasets are not publicly available due to restrictions in the data sharing

agreements with the data sources. The use of de-identified tissue for this study was approved by an Institutional Review Board (IRB).

# Field-specific reporting

nature research | reporting summary

Please select the best fit for your research. If you are not sure, read the appropriate sections before making your selection. Life sciences Behavioural & social sciences Ecological, evolutionary & environmental sciences

For a reference copy of the document with all sections, see nature.com/authors/policies/ReportingSummary-flat.pdf

# Life sciences study design

All studies must disclose on these points even when the disclosure is negative. Sample size

Power calculations using 80% power, 0.05 alpha were used as a guide in determining the the sample size necessary for evaluation.

Data exclusions

Exclusion criteria for validation data were pre-established. Slides were excluded from analysis due to the presence of prostate cancer variants out of the scope of typical Gleason grading, due to artifacts or poor staining that hindered diagnosis, or because of other inability of a genitourinary specialist to confidently assign a diagnosis.

Replication

Not applicable.

Randomization

Allocations of cases into development and validation datasets was random.

Blinding

Annotators were blinded to dataset allocation (development vs. validation) during their reviews.

# Reporting for specific materials, systems and methods

We require information from authors about some types of materials, experimental systems and methods used in many studies. Here, indicate whether each material, system or method listed is relevant to your study. If you are not sure if a list item applies to your research, read the appropriate section before selecting a response.

### Materials & experimental systems Methods

n/a Involved in the study

Unique biological materials Antibodies

Eukaryotic cell lines Palaeontology

Animals and other organisms Human research participants

n/a Involved in the study

ChIP-seq

Flow cytometry

MRI-based neuroimaging

Unique biological materials

Policy information about availability of materials Obtaining unique materials

*Describe any restrictions on the availability of unique materials OR confirm that all unique materials used are readily available from the authors or from standard commercial sources (and specify these sources).*

Antibodies

*Describe all antibodies used in the study; as applicable, provide supplier name, catalog number, clone name, and lot number.*

Antibodies used

Validation

*Describe the validation of each primary antibody for the species and application, noting any validation statements on the manufacturer’s website, relevant citations, antibody profiles in online databases, or data provided in the manuscript.*

## Eukaryotic cell lines

*April 2018*

Policy information about cell lines Cell line source(s)

*State the source of each cell line used.*

Authentication

*Describe the authentication procedures for each cell line used OR declare that none of the cell lines used were authenticated.*

3

Mycoplasma contamination

*Confirm that all cell lines tested negative for mycoplasma contamination OR describe the results of the testing for mycoplasma contamination OR declare that the cell lines were not tested for mycoplasma contamination.*

nature research | reporting summary

Commonly misidentified lines

*Name any commonly misidentified cell lines used in the study and provide a rationale for their use.*

(See ICLAC register)

## Palaeontology

*Provide provenance information for specimens and describe permits that were obtained for the work (including the name of the issuing authority, the date of issue, and any identifying information).*

Specimen provenance

Specimen deposition

*Indicate where the specimens have been deposited to permit free access by other researchers.*

Dating methods

*If new dates are provided, describe how they were obtained (e.g. collection, storage, sample pretreatment and measurement), where they were obtained (i.e. lab name), the calibration program and the protocol for quality assurance OR state that no new dates are provided.*

Tick this box to confirm that the raw and calibrated dates are available in the paper or in Supplementary Information.

## Animals and other organisms

Policy information about studies involving animals; ARRIVE guidelines recommended for reporting animal research Laboratory animals

*For laboratory animals, report species, strain, sex and age OR state that the study did not involve laboratory animals.*

Wild animals

*Provide details on animals observed in or captured in the field; report species, sex and age where possible. Describe how animals were caught and transported and what happened to captive animals after the study (if killed, explain why and describe method; if released, say where and when) OR state that the study did not involve wild animals.*

Field-collected samples

*For laboratory work with field-collected samples, describe all relevant parameters such as housing, maintenance, temperature, photoperiod and end-of-experiment protocol OR state that the study did not involve samples collected from the field.*

## Human research participants

Policy information about studies involving human research participants Population characteristics

*Describe the covariate-relevant population characteristics of the human research participants (e.g. age, gender, genotypic information, past and current diagnosis and treatment categories). If you filled out the behavioural & social sciences study design questions and have nothing to add here, write "See above."*

Recruitment

*Describe how participants were recruited. Outline any potential self-selection bias or other biases that may be present and how these are likely to impact results.*

## ChIP-seq

### Data deposition

Confirm that both raw and final processed data have been deposited in a public database such as GEO. Confirm that you have deposited or provided access to graph files (e.g. BED files) for the called peaks.

Data access links

*For "Initial submission" or "Revised version" documents, provide reviewer access links. For your "Final submission" document, provide a link to the deposited data.*

*May remain private before publication.*

Files in database submission

*Provide a list of all files available in the database submission.*

Genome browser session

*Provide a link to an anonymized genome browser session for "Initial submission" and "Revised version" documents only, to enable peer review. Write "no longer applicable" for "Final submission" documents.*

(e.g. UCSC)

### Methodology

Replicates

*Describe the experimental replicates, specifying number, type and replicate agreement.*

Sequencing depth

*Describe the sequencing depth for each experiment, providing the total number of reads, uniquely mapped reads, length of reads and whether they were paired- or single-end.*

*April 2018*

Antibodies

*Describe the antibodies used for the ChIP-seq experiments; as applicable, provide supplier name, catalog number, clone name, and lot number.*

Peak calling parameters

*Specify the command line program and parameters used for read mapping and peak calling, including the ChIP, control and index files used.*

4

Data quality

*Describe the methods used to ensure data quality in full detail, including how many peaks are at FDR 5% and above 5-fold enrichment.*

nature research | reporting summary

Software

*Describe the software used to collect and analyze the ChIP-seq data. For custom code that has been deposited into a community repository, provide accession details.*

## Flow Cytometry

### Plots

Confirm that:

The axis labels state the marker and fluorochrome used (e.g. CD4-FITC).

The axis scales are clearly visible. Include numbers along axes only for bottom left plot of group (a 'group' is an analysis of identical markers). All plots are contour plots with outliers or pseudocolor plots.

A numerical value for number of cells or percentage (with statistics) is provided.

### Methodology

Sample preparation

*Describe the sample preparation, detailing the biological source of the cells and any tissue processing steps used.*

Instrument

*Identify the instrument used for data collection, specifying make and model number.*

Software

*Describe the software used to collect and analyze the flow cytometry data. For custom code that has been deposited into a community repository, provide accession details.*

Cell population abundance

*Describe the abundance of the relevant cell populations within post-sort fractions, providing details on the purity of the samples and how it was determined.*

Gating strategy

*Describe the gating strategy used for all relevant experiments, specifying the preliminary FSC/SSC gates of the starting cell population, indicating where boundaries between "positive" and "negative" staining cell populations are defined.*

Tick this box to confirm that a figure exemplifying the gating strategy is provided in the Supplementary Information.

## Magnetic resonance imaging

### Experimental design

Design type

*Indicate task or resting state; event-related or block design.*

Design specifications

*Specify the number of blocks, trials or experimental units per session and/or subject, and specify the length of each trial or block (if trials are blocked) and interval between trials.*

Behavioral performance measures

*State number and/or type of variables recorded (e.g. correct button press, response time) and what statistics were used to establish that the subjects were performing the task as expected (e.g. mean, range, and/or standard deviation across subjects).*

### Acquisition

Imaging type(s)

*Specify: functional, structural, diffusion, perfusion.*

Field strength

*Specify in Tesla*

Sequence & imaging parameters

*Specify the pulse sequence type (gradient echo, spin echo, etc.), imaging type (EPI, spiral, etc.), field of view, matrix size, slice thickness, orientation and TE/TR/flip angle.*

Area of acquisition

*State whether a whole brain scan was used OR define the area of acquisition, describing how the region was determined.*

Diffusion MRI Used Not used

### Preprocessing

Preprocessing software

*Provide detail on software version and revision number and on specific parameters (model/functions, brain extraction, segmentation, smoothing kernel size, etc.).*

*April 2018*

Normalization

*If data were normalized/standardized, describe the approach(es): specify linear or non-linear and define image types used for transformation OR indicate that data were not normalized and explain rationale for lack of normalization.*

Normalization template

*Describe the template used for normalization/transformation, specifying subject space or group standardized space (e.g. original Talairach, MNI305, ICBM152) OR indicate that the data were not normalized.*

5

Noise and artifact removal

*Describe your procedure(s) for artifact and structured noise removal, specifying motion parameters, tissue signals and physiological signals (heart rate, respiration).*

nature research | reporting summary

Volume censoring

*Define your software and/or method and criteria for volume censoring, and state the extent of such censoring.*

### Statistical modeling & inference

Model type and settings

*Specify type (mass univariate, multivariate, RSA, predictive, etc.) and describe essential details of the model at the first and second levels (e.g. fixed, random or mixed effects; drift or auto-correlation).*

Effect(s) tested

*Define precise effect in terms of the task or stimulus conditions instead of psychological concepts and indicate whether ANOVA or factorial designs were used.*

Specify type of analysis: Whole brain ROI-based Both

Statistic type for inference

*Specify voxel-wise or cluster-wise and report all relevant parameters for cluster-wise methods.*

(See Eklund et al. 2016)

Correction

*Describe the type of correction and how it is obtained for multiple comparisons (e.g. FWE, FDR, permutation or Monte Carlo).*

### Models & analysis

n/a Involved in the study

Functional and/or effective connectivity Graph analysis

Multivariate modeling or predictive analysis

Functional and/or effective connectivity

*Report the measures of dependence used and the model details (e.g. Pearson correlation, partial correlation, mutual information).*

Graph analysis

*Report the dependent variable and connectivity measure, specifying weighted graph or binarized graph, subject- or group-level, and the global and/or node summaries used (e.g. clustering coefficient, efficiency, etc.).*

Multivariate modeling and predictive analysis

*Specify independent variables, features extraction and dimension reduction, model, training and evaluation metrics.*

This checklist template is licensed under a Creative Commons Attribution 4.0 International License, which permits use, sharing, adaptation, distribution and reproduction in any medium or format, as long as you give appropriate credit to the original author(s) and the source, provide a link to the Creative Commons license, and indicate if changes were made. The images or other third party material in this article are included in the article's Creative Commons license, unless indicated otherwise in a credit line to the material. If material is not included in the article's Creative Commons license and your intended use is not permitted by statutory regulation or exceeds the permitted use, you will need to obtain permission directly from the copyright holder. To view a copy of this license, visit <http://creativecommons.org/licenses/by/4.0/>


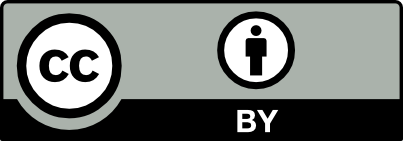


*April 2018*
