# Supplementary material for: Development and validation of a deep learning algorithm for improving Gleason scoring of prostate cancer
Source: NPJ Digit Med. 2019 Jun 7;2:48. doi: 10.1038/s41746-019-0112-2 (PMC6555810; doi:10.1038/s41746-019-0112-2)
Supplement: Supplementary file 2 — Supplemental Materials [file 41746_2019_112_MOESM2_ESM.pdf]

Supplementary Information

Supplementary Table 1: Characteristics of the training and tuning datasets 2
Supplementary Table 2: Description of handling of variants. 3
Supplementary Table 3: Analysis on slides excluded from validation set. 4
Supplementary Table 4: Comparison of DLS to pathologists' unadjusted accuracy. 6
Supplementary Table 5: Comparisons of unadjusted accuracy on the validation set for the 19 pathologists who each reviewed a subset of the validation dataset. 7
Supplementary Table 6: Comparison of DLS to pathologists using other evaluation metrics. 8
Supplementary Table 7: Comparison of %GP 3,4,5 quantitation. 9
Supplementary Table 8: Comparison of %GP4 quantitation in GG2-3 slides and %GP5 quantitation in GG4-5 slides. 10
Supplementary Table 9: Sensitivity analysis excluding consult cases. 11
Supplementary Table 10: Adverse Clinical Event Models Derived From Gleason Pattern Quantitation and Fine-Grained Gleason Pattern Quantitation. 12
Supplementary Fig. 1: Confusion Matrices for the DLS and two pathologist subgroups 13
Supplementary Fig. 2: Model and pathologist concordance with mixed grade labels. 14
Supplementary Fig. 3: Extended visualization of Gleason patterns. 15
Supplementary Fig. 4: Screenshot of the tool used for region-level annotations. 16
Supplementary Fig. 5: Development of datasets used for training, tuning and validation. 17

Supplementary Methods 18
Grading 18
Pathologist Slide-Level Gleason Scoring Protocol 18
Pathologist Region-Level Annotation Protocol 18
Development of the Deep Learning System 19
Hard-Negative Mining 21
Fine-grained Gleason Pattern (GP) 22
Statistical Analysis 22
Comparison with the Cohort-of-29 22
Bootstrap Approach for Confidence Intervals 23

Supplementary Results 23
DLS Region-level Errors 23

Supplementary References 25

Supplementary Table 1: Characteristics of the training and tuning datasets.

		TCGA	Tertiary Teaching Hospital	Medical Laboratory	Total (%)
Number of patients		178	204	7	389
Number of patients excluded due to non-gradable prostate cancer variants, extensive artifacts, or poor staining		43	4	0	47
Number of patients included in the study		135	200	7	342
Number of slides		170	1,016	40	1,226
With slide-level Gleason scores	Number of slides	144	988	27	1,159 (100%)
	GG 1 (slides)	18	558	0	576 (50%)
	GG 2 (slides)	32	218	0	250 (22%)
	GG 3 (slides)	21	84	0	105 (9%)
	GG 4-5 (slides)	73	128	27	228 (20%)
	GG 4	12	25	3	40 (3%)
	GG 5	61	103	24	188 (16%)
With region-level Gleason pattern annotations	Number of slides	148	751	13	912
	Number of patches	14,422,449	97,737,450	455,947	112,615,846 (100%)
	Benign (patches)	11,188,435	93,691,585	364,838	105,244,858 (93%)
	GP3 (patches)	1,335,165	2,131,666	777	3,467,608 (3%)
	GP4 (patches)	1,898,849	1,210,348	67,346	3,176,543 (3%)
	GP5 (patches)	714,666	703,851	22,986	1,441,503 (1%)

Supplementary Table 1: Characteristics of the train and tuning datasets. In these datasets, 1-7 slides from each patient were used, and each slide was reviewed by 3-5 pathologists. Slides were excluded from training/tuning if any pathologist deemed the slide ungradable due to variants or poor image quality. Slide-level Gleason scores and region-level Gleason pattern annotations were collected for overlapping subsets of these slides, with the breakdown described in the table above.

Supplementary Table 2: Description of handling of variants.

**Prostate Cancer Variant**	**Action**
Small Cell Carcinoma	Excluded
Mucinous prostatic adenocarcinoma	Excluded
Adenocarcinoma with signet ring cell like features	Graded via ISUP 2014 recommendations[1]
Prostate ductal adenocarcinoma	Excluded
Basal cell carcinoma	Excluded
**Histological Variant of Acinar Prostatic Adenocarcinoma**	**Action**
Mucinous fibroplasia	Graded via ISUP 2014 recommendations[1]
Foamy gland carcinoma	Graded via ISUP 2014 recommendations[1]
Paneth cell-like neuroendocrine differentiation.	Excluded
Treated prostatic adenocarcinoma	Excluded
Pseudohyperplastic prostatic adenocarcinoma	Graded via ISUP 2014 recommendations[1]
Intraductal carcinoma of the prostate	When found in conjunction with Gleason Gradable tumor, only the Gleason gradable component is graded (consistent with ISUP 2014 recommendations)[1]

Supplementary Table 2: Description of handling of prostate cancer variants and acinar adenocarcinoma histological variants. Slides containing cancer variants and histological variants that are not Gleason gradable were excluded from the study (with the exception of intraductal carcinoma). Other variants are graded in a manner consistent with ISUP 2014 recommendations.

Supplementary Table 3: Analysis on slides excluded from validation set.

Slide	Rationale for lack of confidence in diagnosis	Specialist 1 GG	Specialist 2 GG	Specialist 3 GG	DLS GG
1	need IHC - high grade tumor, but needs IHC to assess/quantify IDC vs pattern 5	4-5	4-5	4-5	4-5
2	need IHC - 4+3 vs 4+5 (pattern 5 based on cribriform necrosis), but chatter artifact makes it difficult to tell; also would do IHC to r/o IDC vs pattern 4/5 areas	3	3	3	4-5
3	need IHC - high grade tumor case, but with areas of IDC vs pattern 4 vs pattern 4 with necrosis (pattern 5)	3	4-5	3	4-5
4	need IHC - areas of IDC vs pattern 4 vs pattern 4 with necrosis (pattern 5)	2	3	2	2
5	need IHC - likely 4+3 case, but given prominent areas of possible HGPIN/IDC need IHC to accurately quant pattern 4	3	3	3	3
6	need IHC - given large areas of large cribriform glands (DDx HGPIN/IDC vs pattern 4), need IHC to accurately quantitate and grade	4-5	4-5	4-5	4-5
7	need IHC - large areas of possible IDC; need IHC to r/o vs pattern 4 and for accurate pattern 4/tumor vol %	3	3	3	3
8	need IHC - focal area of large cribriform glands present, would do both stains (r/o IDC vs pattern 4) and also levels as there may be necrosis (IDC vs pattern 5)	2	2	2	2
9	need IHC - definite invasive cancer present, but adjacent large cribriform glands with DDx of pattern 4 vs HGPIN needs IHCs to assess/quantitate	3	3	3	3
10	need IHC - areas of large cribriform glands needing IHC to eval IDC vs pattern 4	3	3	3	3
11	need IHC - areas of definite large crib irregular glands of pattern 4, but some areas of probable IDC also (need IHC to accurately quantitate)	4-5	4-5	4-5	4-5
12	Needs another expert review. There is a pattern 3 that is not recognized. I don’t see a pattern 5.	3	3	3	4-5
13	I suggest this case go to another expert as this case has many patterns and is good to our criteria titrated	3	4-5	3	4-5
14	No agreement among initial reviewers - suggest another expert opinion	4-5	3	3	2
15	Show to colleague(s) and order serial sections to confirm small % GG4	2	2	2	2
16	Challenging slide - perhaps tissue was not well fixed as morphology was not great for grading. As such, I am not sure about GG5 - would show to a colleague(s).	4-5	3	3	4-5
17	Show to colleague(s) and order serial sections to confirm small % GG4	2	2	2	1
18	Challenging case - would order serial sections to confirm minor GG5 (rule out tangential sectioning of GG4 poorly formed acini) as well as show to a colleague	4-5	4-5	3	4-5
19	require IHC	3	3	2	3
20	Would use basal cell IHC to rule in/rule out intraductal carcinoma	3	3	2	4-5

Supplementary Table 3: Analysis on slides excluded from validation set due to genitourinary specialist lack of confidence when diagnosing. 20 slides were excluded from the analysis in the main text where the specialist adjudicator was not able to provide a confident diagnosis. Consults were subsequently provided by the other two GU experts. Of the 12 cases where the original adjudicator and two consulting experts came to a consensus, the DLS was concordant on 9 (highlighted in green) and within 1 grouping on the remaining 3 (highlighted in red).

Supplementary Table 4: Comparison of DLS to pathologists' unadjusted accuracy.

Grader	Unadjusted accuracy for grade group (95% CI)	p-value for comparison with DLS	Years since anatomic pathology residency	Reported monthly prostate case volume
Deep learning system	0.698 (0.650, 0.746)	n/a		
Mean among all 29 pathologists	0.610 (0.563, 0.660)	0.002		
Mean among 19-pathologist subgroup	0.596 (0.529, 0.659)	<0.001		
Mean among 10-pathologist subgroup (A-J below)	0.637 (0.588, 0.686)	0.006		
Pathologist A	0.526 (0.468, 0.577)	<0.001	9	≤10
Pathologist B	0.559 (0.502, 0.613)	<0.001	6	Not reported
Pathologist C	0.592 (0.538, 0.644)	<0.001	4	≤10
Pathologist D	0.628 (0.574, 0.680)	0.027	10	≤10
Pathologist E	0.647 (0.592, 0.695)	0.16	3	10-20
Pathologist F	0.640 (0.589, 0.689)	0.083	1	>20
Pathologist G	0.668 (0.616, 0.716)	0.40	4	Not reported
Pathologist H	0.671 (0.616, 0.722)	0.45	18	≤10
Pathologist I	0.716 (0.668, 0.764)	0.59	26	>20
Pathologist J	0.728 (0.683, 0.776)	0.33	16	>20

Supplementary Table 4: Comparison of unadjusted concordance between the deep learning system, the cohort of 29 pathologists, and 10 individual pathologists (A-J). The cohort of 29 pathologist comprised of 10 pathologists (A-J) that reviewed all 331 slides in the validation dataset and 19 pathologist that each reviewed a subset of the validation dataset. For the concordance of the individual 19 pathologists see Supplementary Table 5. Confidence intervals (CIs) were calculated with 1000

bootstrap replications. The statistical significance of the comparisons were performed using the permutation test.

Supplementary Table 5: Comparisons of unadjusted accuracy on the validation set for the 19 pathologists who each reviewed a subset of the validation dataset.

Grader	Number of slides in subset	Pathologist accuracy on subset (95% CI)	DLS accuracy on subset (95% CI)	Years since anatomic pathology residency	Reported monthly prostate case volume
Pathologist K	62	0.306 (0.194, 0.435)	0.742 (0.629, 0.840)	3	≤10
Pathologist L	64	0.422 (0.312, 0.555)	0.672 (0.570, 0.774)	2	>20
Pathologist M	55	0.545 (0.400, 0.655)	0.618 (0.500, 0.746)	28	≤10
Pathologist N	58	0.552 (0.414, 0.655)	0.603 (0.483, 0.716)	20	10-20
Pathologist O	54	0.556 (0.444, 0.648)	0.630 (0.519, 0.759)	19	Not reported
Pathologist P	57	0.561 (0.439, 0.684)	0.789 (0.675, 0.877)	20	10-20
Pathologist Q	49	0.571 (0.438, 0.694)	0.694 (0.592, 0.807)	22	10-20
Pathologist R	40	0.575 (0.450, 0.725)	0.700 (0.575, 0.850)	37	10-20
Pathologist S	50	0.580 (0.440, 0.730)	0.700 (0.600, 0.800)	24	10-20
Pathologist T	50	0.580 (0.460, 0.690)	0.700 (0.599, 0.850)	3	10-20
Pathologist U	53	0.623 (0.500, 0.736)	0.642 (0.528, 0.774)	4	Not reported
Pathologist V	49	0.633 (0.510, 0.755)	0.673 (0.550, 0.786)	3	10-20
Pathologist W	57	0.649 (0.509, 0.772)	0.737 (0.622, 0.851)	11	Not reported
Pathologist X	60	0.650 (0.500, 0.750)	0.700 (0.600, 0.800)	14	Not reported
Pathologist Y	46	0.674 (0.543, 0.783)	0.652 (0.510, 0.761)	1	Not reported
Pathologist Z	44	0.682 (0.500, 0.818)	0.705 (0.579, 0.818)	6	Not reported
Pathologist AA	50	0.700 (0.560, 0.820)	0.700 (0.540, 0.830)	16	≤10

Pathologist AB	41	0.732 (0.610, 0.854)	0.732 (0.597, 0.878)	14	Not reported
Pathologist AC	53	0.736 (0.623, 0.887)	0.698 (0.584, 0.821)	2	10-20

Supplementary Table 5: **Comparisons of unadjusted accuracy on overlapping subsets of the validation set for the cohort of 19 pathologists.** Each pathologist reviewed a subset of the validation dataset, that collectively provided 3 annotations per slide for each of the 331 validation slides. In this subgroup analysis, the DLS's accuracy is greater than that of 14 of the 19 pathologists.

Supplementary Table 6: Comparison of DLS to pathologists using other evaluation metrics.

Grader	Population-adjusted accuracy for grade group (95% CI)	p-value for comparison with DLS	Cohen's kappa for grade group (95% CI)	p-value for comparison with DLS	Accuracy for Gleason score (6-10) (95% CI)	p-value for comparison with DLS
Deep learning system	0.720 (0.675, 0.762)	n/a	0.585 (0.520, 0.651)	n/a	0.770 (0.722, 0.813)	n/a
Mean among all 29 pathologists	0.628 (0.578, 0.674)	<0.001	0.466 (0.398, 0.527)	0.001	0.681 (0.638, 0.725)	<0.001
Pathologist A	0.515 (0.459, 0.569)	<0.001	0.365 (0.290, 0.430)	<0.001	0.672 (0.623, 0.723)	0.002
Pathologist B	0.572 (0.519, 0.625)	<0.001	0.412 (0.341, 0.481)	<0.001	0.593 (0.540, 0.646)	<0.001
Pathologist C	0.615 (0.565, 0.660)	<0.001	0.457 (0.389, 0.522)	0.001	0.703 (0.651, 0.752)	0.039
Pathologist D	0.679 (0.635, 0.720)	0.16	0.489 (0.415, 0.556)	0.018	0.659 (0.607, 0.710)	<0.001
Pathologist E	0.603 (0.549, 0.655)	0.003	0.506 (0.428, 0.573)	0.10	0.734 (0.689, 0.777)	0.27
Pathologist F	0.634 (0.581, 0.685)	0.011	0.514 (0.441, 0.577)	0.088	0.729 (0.683, 0.777)	0.19
Pathologist G	0.656 (0.605, 0.712)	0.070	0.530 (0.459, 0.600)	0.21	0.734 (0.686, 0.782)	0.25
Pathologist H	0.669 (0.618, 0.721)	0.12	0.548 (0.475, 0.618)	0.37	0.690 (0.638, 0.736)	0.007
Pathologist I	0.727 (0.679, 0.775)	0.81	0.613 (0.548, 0.678)	0.45	0.769 (0.720, 0.815)	>.99
Pathologist J	0.758 (0.714, 0.801)	0.18	0.622 (0.561, 0.690)	0.33	0.773 (0.727, 0.818)	>.99

Supplementary Table 6: Comparison of other evaluation metrics (adjusted accuracy for grade group, Cohen's Kappa for grade group, and accuracy for Gleason score) between the deep learning system (DLS), the cohort of 29 pathologists, and 10 individual pathologists (A-J). The adjusted accuracy reflects a population-level GG distribution of 7397:8353:3106:1968.[2] Confidence intervals (CIs) were calculated with 1000 bootstrap replications. The statistical significance of the comparisons were performed using the permutation test.

Supplementary Table 7: Comparison of %GP 3,4,5 quantitation.

Grader	Mean absolute error for %GP3 (95% CI)	p-value for comparison with DLS	Mean absolute error for %GP4 (95% CI)	p-value for comparison with DLS	Mean absolute error for %GP5 (95% CI)	p-value for comparison with DLS
Deep learning system	11.9 (10.0, 13.9)	n/a	11.8 (10.5, 13.2)	n/a	4.5 (3.4, 5.7)	n/a
Mean among all 29 pathologists	16.0 (13.3, 18.7)	0.004	17.8 (15.1, 20.7)	<0.001	5.2 (3.9, 6.6)	0.26
Pathologist A	19.4 (16.9, 21.8)	<0.001	22.0 (19.3, 24.7)	<0.001	4.2 (3.0, 5.4)	0.43
Pathologist B	19.5 (17.0, 22.3)	<0.001	22.6 (19.8, 25.5)	<0.001	5.4 (3.9, 6.9)	0.19
Pathologist C	18.5 (15.9, 21.1)	<0.001	21.0 (18.3, 23.9)	<0.001	4.2 (3.1, 5.6)	0.49
Pathologist D	13.1 (11.3, 14.9)	0.36	15.8 (13.9, 17.7)	<0.001	5.0 (3.7, 6.5)	0.50
Pathologist E	15.6 (13.7, 17.6)	0.002	18.8 (16.8, 20.7)	<0.001	4.3 (3.3, 5.6)	0.80
Pathologist F	15.2 (13.0, 17.4)	0.002	17.0 (14.7, 19.3)	<0.001	4.9 (3.6, 6.2)	0.51
Pathologist G	10.4 (9.1, 12.0)	0.19	14.6 (12.8, 16.5)	0.001	6.9 (5.5, 8.4)	<0.001
Pathologist H	10.2 (8.8, 11.7)	0.12	14.5 (12.5, 16.3)	0.003	6.5 (4.9, 8.2)	<0.001
Pathologist I	9.8 (8.3, 11.2)	0.083	11.9 (10.3, 13.4)	>0.99	4.2 (3.2, 5.5)	0.55
Pathologist J	10.2 (8.6, 11.8)	0.13	12.2 (10.5, 14.0)	0.68	3.9 (2.9, 5.0)	0.10

Supplementary Table 7: Comparison of Gleason pattern (GP) quantitation between the deep learning system (DLS), the cohort of 29 pathologists, and 10 individual pathologists. Confidence intervals (CIs) were calculated with 1000 bootstrap replications. The statistical significance of the comparisons were performed using the permutation test.

Supplementary Table 8: Comparison of %GP4 quantitation in GG2-3 slides and %GP5 quantitation in GG4-5 slides.

Grader	Mean absolute error for %GP4 in GG 2-3 slides (95% CI)	p-value for comparison with DLS	Mean absolute error for %GP5 in GG 4-5 slides (95% CI)	p-value for comparison with DLS
Deep Learning System	13.0 (11.5, 14.7)	n/a	18.7 (14.3, 23.2)	n/a
Mean among all 29 pathologists	20.5 (17.6, 24.0)	<0.001	22.0 (18.0, 26.9)	0.30
Pathologist A	27.3 (23.8, 30.8)	<0.001	18.0 (13.5, 23.2)	0.76
Pathologist B	25.1 (21.7, 28.7)	<0.001	24.7 (19.3, 30.4)	0.076
Pathologist C	25.5 (22.1, 28.9)	<0.001	19.6 (14.0, 25.4)	0.79
Pathologist D	19.1 (16.6, 21.7)	<0.001	24.2 (19.0, 29.6)	0.12
Pathologist E	19.3 (17.0, 21.7)	<0.001	20.6 (16.1, 25.0)	0.58
Pathologist F	18.0 (15.5, 20.6)	<0.001	19.0 (13.9, 24.3)	0.89
Pathologist G	16.1 (14.0, 18.3)	0.007	20.9 (15.7, 26.3)	0.35
Pathologist H	15.4 (13.2, 17.6)	0.044	24.0 (18.2, 31.0)	0.046
Pathologist I	13.9 (12.0, 15.8)	0.38	17.5 (12.8, 22.7)	0.49
Pathologist J	14.6 (12.6, 16.8)	0.12	17.4 (13.1, 22.0)	0.53

Supplementary Table 8: Comparison of Gleason pattern (GP) in Grade Groups (GG) 2-3 and 4-5 between the deep learning system (DLS), the cohort of 29 pathologists, and 10 individual pathologists (A-J). Confidence intervals (CIs) were calculated with 1000 bootstrap replications. The statistical significance of the comparisons were performed using the permutation test.

Supplementary Table 9: Sensitivity analysis excluding consult cases.

Grader	Number of Slides excluded due to indication of a non-confident diagnosis	Pathologist's accuracy excluding consult cases (95% CI)	DLS accuracy excluding the same cases (95% CI)	p-value for comparison with DLS
Pathologist A	3	52.0 (46.7, 57.3)	69.2 (64.2, 74.1)	<0.001
Pathologist B	6	56.2 (50.6, 61.8)	69.6 (64.8, 74.7)	<0.001
Pathologist C	2	59.9 (54.2, 65.3)	69.4 (64.7, 74.3)	0.002
Pathologist D	10	63.3 (58.0, 68.5)	69.8 (65.0, 74.4)	0.048
Pathologist E	3	64.7 (59.7, 69.7)	69.3 (64.5, 74.4)	0.22
Pathologist F	7	63.7 (58.4, 68.6)	69.5 (64.7, 74.5)	0.074
Pathologist G	2	67.0 (62.0, 72.2)	69.5 (64.7, 74.4)	0.50
Pathologist H	8	66.5 (61.5, 71.8)	69.6 (64.8, 74.4)	0.37
Pathologist I	9	71.4 (66.9, 76.0)	69.6 (64.5, 74.4)	0.60
Pathologist J	1	73.8 (69.1, 78.6)	69.5 (64.6, 74.6)	0.17

Supplementary Table 9: Comparison between pathologists and DLS on Gleason scoring excluding slides indicated by pathologists as non-confident diagnosis. The results are qualitatively similar to the results in Supplementary Table 4 with no material differences. Confidence intervals (CIs) were calculated with 1000 bootstrap replications. The statistical significance of the comparisons were performed using the permutation test.

Supplementary Table 10: Adverse Clinical Event Models Derived From Gleason Pattern Quantitation and Fine-Grained Gleason Pattern Quantitation.

Source of Gleason pattern quantitation	Input features to Cox regression model describing tumor composition: (all based on % Gleason pattern)	C-index (95% CI)
Cohort-of-29 general pathologists	3, 4, 5	0.674 (0.564, 0.782)
Genitourinary specialist pathologists	3, 4, 5	0.690 (0.582, 0.800)
DLS	3, 4, 5	0.697 (0.579, 0.790)
DLS	3, 3.5, 4, 5	0.704 (0.586, 0.814)
DLS	3, 3.5, 4, 4.5, 5	0.702 (0.577,0.812)

Supplementary Table 10: Comparison of Cox models for adverse clinical events (progression/biochemical recurrence) trained directly on quantified Gleason patterns and fine-grained Gleason Patterns. Cox proportional hazards regression models were trained and evaluated on the validation set (n=331 slides), with Gleason patterns quantitation as input features. Features were provided by the cohort-of-29 pathologists, genitourinary specialists comprising the reference standard, and the DLS. As proof-of-concept, Cox models were also trained with additional features that provide finer-grained representations of tumor differentiation (see “Fine-grained Gleason Pattern” in Supplementary Methods). Confidence intervals (CI) were calculated via bootstrapping, and the median concordance index is presented for the cohort-of-29 pathologists (see Supplementary Methods).

Supplementary Fig. 1: Confusion Matrices for the DLS and two pathologist subgroups

Specialist-Adjudicated Grade Group vs. Deep Learning System's Grade Group (n=331 slides)

Specialist-Adjudicated Grade Group	GG1	GG2	GG3	GG4-5
GG1 (n=77 slides)	90%	10%	0%	0%
GG2 (n=134 slides)	22%	66%	10%	1%
GG3 (n=62 slides)	3%	21%	34%	42%
GG4-5 (n=58 slides)	2%	2%	7%	89%

Subgroup of 10 pathologists (n=331 slides with 10 annotations/slide)

Specialist-Adjudicated Grade Group	GG1	GG2	GG3	GG4-5
GG1 (n=77 slides)	73%	23%	3%	2%
GG2 (n=134 slides)	20%	58%	16%	5%
GG3 (n=62 slides)	1%	18%	49%	32%
GG4-5 (n=58 slides)	1%	4%	16%	79%

Subgroup of 19 pathologists (n=331 slides with 3 annotations/slide)

Specialist-Adjudicated Grade Group	GG1	GG2	GG3	GG4-5
GG1 (n=77 slides)	79%	15%	5%	1%
GG2 (n=134 slides)	31%	51%	14%	4%
GG3 (n=62 slides)	8%	33%	38%	21%
GG4-5 (n=58 slides)	1%	6%	21%	72%

Supplementary Fig. 1: Confusion matrices highlighting the distribution of errors made by the DLS and two pathologist subcohorts. The DLS is compared to the subgroup of 10 pathologists where each pathologist individually annotated every validation set slide, as well as the subgroup of 19 pathologists that collectively provided 3 reviews for every slide. The DLS shows greater accuracy in classifying slides as GG1, GG2, and GG4-5, and lower accuracy in classification of GG3 on the validation set as compared to these cohorts.

Supplementary Fig. 2: Model and pathologist concordance with mixed grade labels.

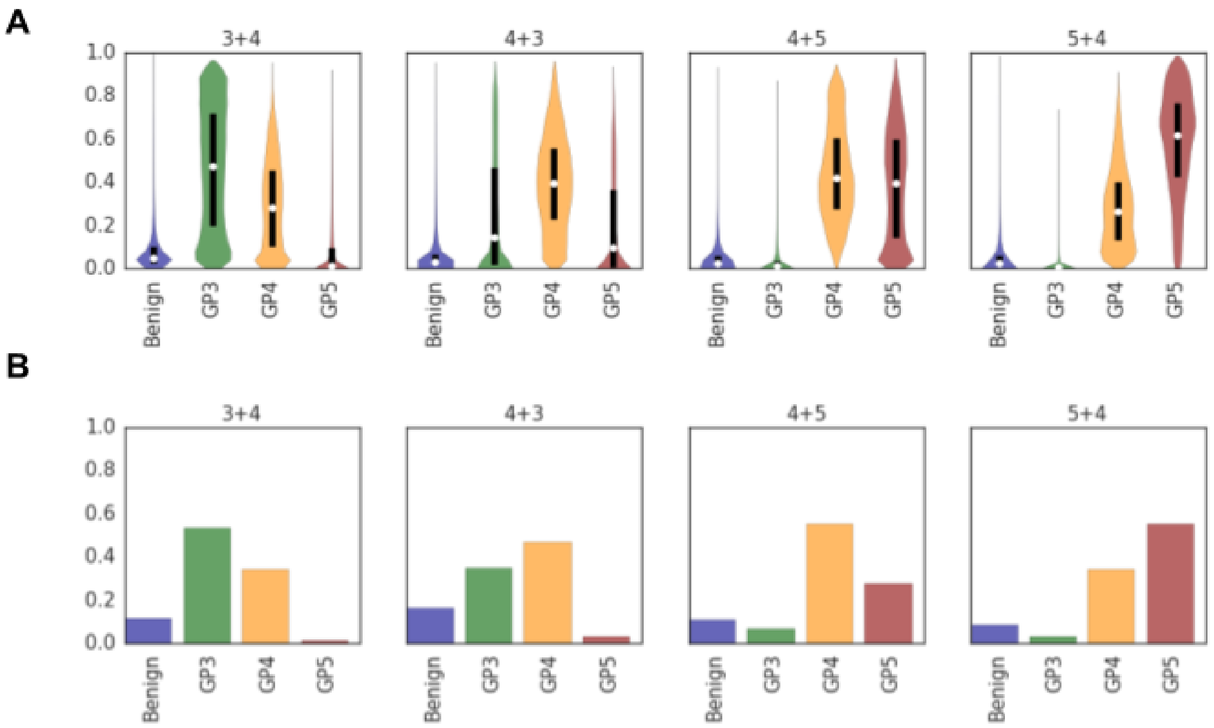

Supplementary Fig. 2: Model and pathologist concordance with mixed grade labels. When pathologists could not assign a single Gleason pattern to a region, they were instructed to assign a mixed grade label. Available mixed grade labels were ‘3+4’, ‘4+3’, ‘4+5’, and ‘5+4’. These indicate that a region exhibits histological patterns characteristic of both Gleason patterns at the level of glands, and they are an extension to the Gleason grading system which allow humans to represent a small slice of the continuum of Gleason grading. To further investigate the deep learning system’s ability to quantitatively represent the ambiguities present in the Gleason grading system, we examine the model’s output in those cases in which a pathologist provided a mixed grade. **A,** Distributions of predicted likelihood of each GP by the DLS on patches labeled as a mixed grade by at least one pathologist. The DLS represents “in-between” patterns by exhibiting mixed likelihood between multiple labels. **B,** The distribution of other pathologist grades for those patches which were given a mixed grade by at least one pathologist.

Supplementary Fig. 3: Extended visualization of Gleason patterns.

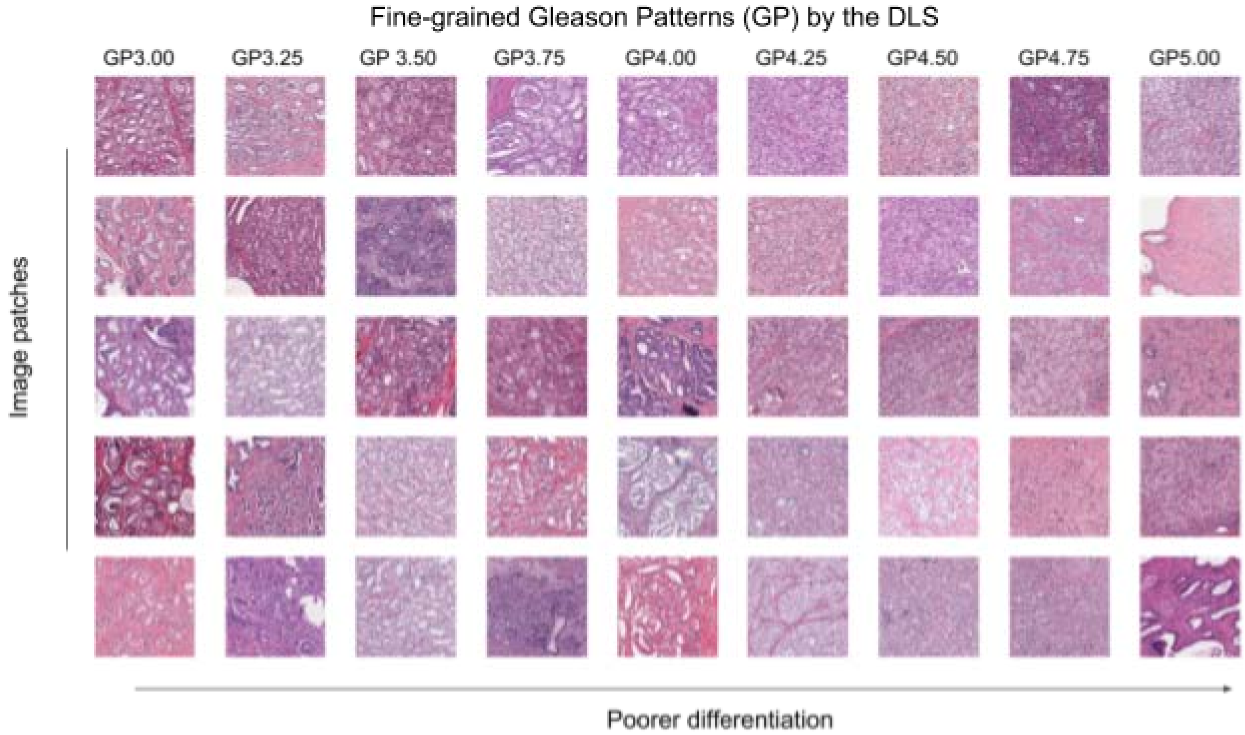

Supplementary Fig. 3: Extended visualization of Gleason patterns. The continuum of prostate cancer Gleason Patterns (GP) learned by the DLS reveals finer categorization of the well-to-poorly differentiated spectrum. The top row highlights the DLS GP categorization followed by H&E images that are predicted to be the corresponding quantitative GP. Columns 1, 5, and 9 represent 100% confidence in GP 3, 4, and 5 respectively. The columns in between represent quantitative GPs that are in between these defined categories.

Supplementary Fig. 4: Screenshot of the tool used for region-level annotations.

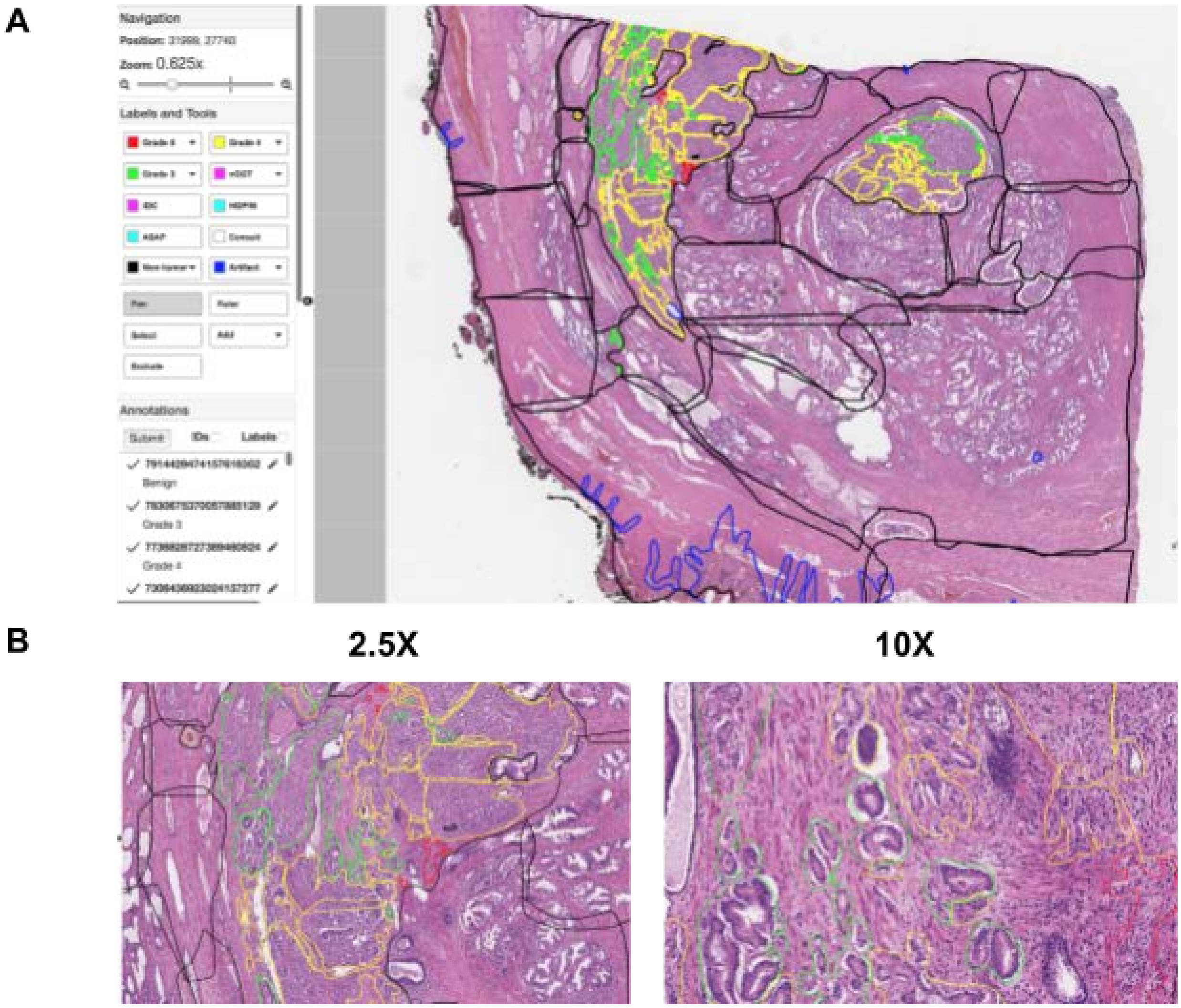

Supplementary Fig. 4: Screenshots of the tool used for region-level annotations. A, An overview of the tool zoomed out to 0.625X. A user annotates a region by first selecting a label category on the left and then outlining the corresponding regions direct on the slide. This custom free-hand drawing tool also has the ability to zoom between different objective powers as appropriate. **B,** Screenshots of annotations on tissue regions at additional magnifications: 2.5X and 10X. Most annotations were done between 5-20X.

Whole Slide Images

	Training	Tuning	Validation
Region-Level annotations	1 pathologist per slide	3 pathologists per slide:<br><br>Image patches where ≥2 pathologists were concordant were used	3 pathologists per slide:<br><br>Analysis on patches where all 3 pathologists provided a categorization
Slide-Level annotations	≥3 pathologist reviews:<br><br>Most common grade with ties in favor of the more severe grade for sensitivity	≥3 pathologist initial reviews, and final grade provided by a genitourinary expert	≥3 pathologist initial reviews, and final grade provided by a genitourinary expert

Supplementary Fig. 5: Development of datasets used for training, tuning, and validation. Region-level datasets and the slide-level training datasets were provided by pathologists, while the generation of the slide-level tuning and validation datasets involved genitourinary expert pathologists. More details can be found in the Grading sections of the Methods and the Supplementary Methods.

Supplementary Methods

Grading

Pathologist Slide-Level Gleason Scoring Protocol

Slides used for training were reviewed by at least 3 and up to 7 pathologists (median 4). The label for each slide was determined by the most common annotation provided by the pathologists, while breaking ties in favor of the more severe grade to encourage higher DLS sensitivity. Tuning slides were initially reviewed by 3 to 5 pathologists and subsequently adjudicated by 1 of 3 genitourinary specialists (similar to the validation dataset).

We derived the slide-level Gleason score (e.g. 3+4) from the predominant GP and next-most-common GP. This is used instead of the directly provided Gleason scores because we noted inconsistent application of tertiary replacement (replacing the secondary Gleason score with ‘5’ if %GP5 is greater than 5%), leading to even greater diagnostic variability.[2] The GG (e.g. GG2) was then directly determined using the Gleason score using the published definitions.[2] Pathologists were additionally instructed to note if a slide contained histologic variants (listed in Supplementary Table 2), did not contain tumor, or if they were not confident in their diagnosis.

Pathologist Region-Level Annotation Protocol

The region-annotations for all datasets (training, tuning, and validation) were performed using custom free-hand drawing tools in a custom histopathology viewer (see Supplementary Fig. 4) with the ability to zoom between magnifications. Most annotations were performed between 5X and 20X magnifications. Artifacts that affected the ability to make a confident interpretation were labeled as artifacts, and regions where the pathologists were not able to assign confident categorizations based on their best clinical judgement were assigned a “consult” label. Regions where different GPs were either ambiguous or difficult to delineate exactly were assigned mixed-grade labels such as ‘3+4’. Perineural and lymphovascular invasive tumor and intraductal carcinoma were labeled as non-Gleason-gradable tumors.

For the training slides, at least one pathologist non-exhaustively annotated characteristic regions of each slide (annotated tissue for each slide <1% to 100%, median of 57%). For the tuning slides, we obtained higher-confidence labels by asking three pathologists for exhaustive annotations. In this set, to improve annotation efficiency (retaining slide-level diversity while reducing the overall annotation workload), the pathologists annotated only a subset of each slide, specifically two 3.8x3.8mm square regions from each quadrant on the slide. The locations of the two squares within each quadrant were randomly selected, and all three pathologists annotated the same eight regions (annotated tissue for each slide <1% to 35%, median of 14%). Only image patches with concordance between at least two annotators were used.

To train the stage-1 DLS, we processed the training dataset annotations to retain only regions with unambiguous labels. Ambiguity arising from multiple different labels were resolved by majority vote. Regions labeled ‘artifact’ were interpreted as non-tumor to reduce false positive predictions on artifact-containing regions. Regions labeled as ‘mixed-grade’ were interpreted as the primary pattern (*e.g.*, ‘5+4’ was interpreted as GP5), based on empirical observations of a resultant boost in stage-1 region-level accuracy. For the tuning datasets, only regions for which all three annotators provided a label were considered (similar to the validation dataset). In the main text, we report results only for patches labeled non-tumor, GP3, GP4, GP5. The analysis of image patches that are labeled with mixed-grades are presented in Supplementary Fig. 2.

Development of the Deep Learning System

We used a Inception-V3[3] image classification network, with fewer filters per layer (depth_multiplier=0.1) and modified to be fully-convolutional to improve inference throughput on whole-slide images (manuscript under review). To avoid introducing grid artifacts, the fully-convolutional modification involved using ‘VALID’ instead of ‘SAME’ padding in convolutions and differential cropping of the output of ‘branches’ in the Inception architecture. This network takes as input image patches of size 911x911 pixels at 10X magnification (equivalent to 911 $\times$ 911 μm). The region “assessed” by the network is a 32 $\times$ 32 μm region centered in each image patch.

The training process involved feeding image patches into the network with a specific sampling strategy to avoid bias towards specific slides or classes: first select a class according to the ratios 4:2:2:1

for the four classes respectively, then select a slide containing regions labeled as that class, and finally select an image patch from that slide. To help improve generalization performance, we applied data augmentation techniques to randomly perturb the actual images seen by the neural network (image perturbations for saturation, contrast, brightness, hue, and orientation) during training.[4] Training was performed in TensorFlow[5] using an RMSProp optimizer[6] and the softmax cross-entropy loss function. Hyperparameters such as the four-class sampling ratios, magnitude of image perturbations, the learning rate decay schedule, and L2 regularization decay were tuned via Gaussian-Bandit search on *Google Vizier*.[7] After tuning model hyperparameters, hard negative mining and ensembling were employed to further improve model performance. See below section for details of hard-negative mining.

After model convergence (as determined by the patch-level four-way classification performance on the tuning set, as measured by Cohen's kappa), we applied ensembling at three levels. First, the actual network weights used were smoothed using an exponential moving average with decay constant of 0.9999. Second, for each patch, the model predictions across eight image orientations (4 90° rotations and 2 left-right flips) were averaged using the geometric mean. Lastly, these orientation-averaged predictions were again averaged across four independently trained models (each with a separate hard-negative mining process), again using the geometric mean.

In the second stage of the DLS, we first calibrated each region's class predicted likelihoods. The calibration weights were determined empirically to produce the best slide-level predictions on the tuning set. Next, to obtain a categorical prediction for each patch, we applied the argmax function. Finally, each slide's patch-level predictions were summarized as four features: %Tumor, %GP3, %GP4, and %GP5. We linearly rescaled these features to have a minimum of 0 and a maximum of 1 in the training set, and trained a k-nearest neighbor (kNN) model for each prediction task: 4-way GG classification (GG 1, 2, 3 or 4-5), and each of the three binary classifications of GG $\geq$ 2, GG $\geq$ 3, and GG $\geq$ 4. The hyperparameter "k" (number of nearest neighbors) and neighbor-weighting method (uniform versus reciprocal of distance) were selected based on the performance of each model on the tuning set, as measured by kappa for GG and area under receiver operating characteristic (AUC) for the binary predictions. Our final selected hyperparameters were k=24 with uniform neighbor weighting. In addition, we evaluated the performance of several other machine learning algorithms, such as logistic regression, and random forest on the tuning

set. kNN was selected to avoid over-fitting based on the limited size of the slide-level dataset and for ease of interpretability (as visualized in Fig. 1).

Hard-Negative Mining

Our DLS stage-1 development process includes large scale, continuous “hard-negative mining” which aims to improve algorithm performance by running inference on the entire training dataset to isolate the hardest examples and further refine the algorithm using these examples.

In hard negative mining, inference was run hourly by applying the partially-trained network to the entire training dataset (over 112 million image patches) for the entire duration of the training. These inference results were then used to alter the patch-sampling probabilities for every slide in the training set. For a given class in each slide, these sampling probabilities were initialized at the start of training to be uniform across all image patches. After every inference round, the sampling probabilities were updated to be proportional to the cross-entropy loss of each patch, such that incorrect classifications were sampled more frequently. In other words, as training proceeded, the DLS learned from harder and harder examples, which improved its accuracy more efficiently than random examples. While previous works employing deep learning on histopathology images have employed hard negative mining in an offline “batch-mode”[8–10], we observed that performance improves with the frequency of inference on the entire training dataset, resulting in the “quasi-online” hard-negative mining approach (>30,000 DLS stage-1 inferences per second) used here. We anticipate that the benefits of this continuous hard negative mining approach may be applicable to developing other deep learning algorithms on histopathology images as well.

Fine-grained Gleason Pattern (GP)

To provide a more quantitative GP that smoothly interpolates between existing GPs (3, 4, and 5), we processed the calibrated DLS-predicted likelihood for each GP. First, the predictions for the two GPs with highest confidences were used to interpolate between the two GPs using the formula $likelihood_1$ / ($likelihood_1$ + $likelihood_2$). For example, if the GP 3,4,5 predictions were [0.7, 0.2, 0.1], then the computed value was 0.7 / (0.7 + 0.2) = 0.78, and the quantitative GP was 3+0.78 = 3.78. To visualize these quantitative GPs (e.g. in Fig. 4a), we used the International Commission on Illumination “Lab” (CIELAB)

color space, which is designed to be perceptually uniform with respect to the underlying numerical values. To select regions that represent desired quantitative GPs (Fig. 4c and Supplementary Fig. 3), we located the image patches among all validation dataset slides for which the computed quantitative GP most closely matched the desired GP (e.g. 3.5).

Statistical Analysis

Comparison with the Cohort-of-29

Comparison of the DLS with the cohort-of-29 pathologists required a modified permutation test[11] to account for the different numbers of slide-level annotations provided by each pathologist. Specifically, 10 pathologists annotated all the slides (331 annotations each), while 19 pathologists collectively annotated all the slides 3 times (about 50±10 annotated slides by each pathologist). The 10 pathologists that annotated all the slides were selected based on slide reviewing speed and availability. To represent each pathologist equally, we modify the permutation test as follows: define our test statistic as the difference between the DLS accuracy and the mean accuracy among pathologists in the cohort-of-29. In each iteration of the permutation test, for each slide, randomly swap the model's given rating with one of the 14 ratings given for that slide (allowing the model to "swap" with itself with probability 1/14), and compute the test statistic on the result. After 5000 iterations, this gives a null distribution of the test statistic against which we compare the observed difference to compute a two-tailed p value.

In the risk stratification analyses, the cohort-of-29 pathologists annotations were sampled to approximate equal representation of each pathologist. For each slide, the sampled annotation can come from either one of subgroup-of-10 annotations or one of the 3 available subgroup-of-19 annotations. Specifically, for each slide, an annotation was selected from one of the 10 available subgroup-of-10 annotations with 1/29 probability, or from one of the 3 available subgroup-of-19 annotations with (19/29)*(1/3) probability.

Bootstrap Approach for Confidence Intervals

To compute confidence intervals for the pools of 10, 19, and 29, we bootstrapped both slides and annotators by resampling both with replacement in each iteration of the bootstrap. In the case of the pool

of 29, to replicate our experimental design in each iteration, we separately resampled the subsets of 10 and 19.

Supplementary Results

DLS Region-level Errors

Here, we present a qualitative analysis of the errors made by the DLS's first stage, at the region level. Several errors were related to spatial localization. For example, the spatial extent of each predicted Gleason pattern region was sometimes imprecise; if two tumor-containing regions were separated by a small strip of non-tumor tissue, the DLS would sometimes categorize the intervening non-tumor as tumor.

Similarly, delineating the precise stroma-tumor interface was difficult for the DLS, in particular for GP5 and stroma (non-tumor). This was likely because GP5 can present as individual tumor cells in a background of connective tissue, and outlining each individual cell was impractical. The "impurity" of the underlying region-level annotation made it difficult to develop a DLS that was precision with respect to the boundary.

In many other cases, the errors made by the DLS was one where the underlying histology was ambiguous, such as when a tangential cut into a GP3 region caused it to resemble the fused-gland pattern that defines GP4. Because the DLS was trained to interpret the image patch surrounding the region, it will not take into account context from beyond its input image.

The remaining region-level errors involved true prediction mistakes that will naturally improve with more data. The second stage of the DLS is fairly robust against all of these errors by summarizing the predictions from all regions on the slide as a small number of features.

Supplementary References

1. Epstein, J. I. *et al.* The 2014 International Society of Urological Pathology (ISUP) Consensus Conference on Gleason Grading of Prostatic Carcinoma: Definition of Grading Patterns and Proposal for a New Grading System. *Am. J. Surg. Pathol.* **40,** 244–252 (2016).
2. Epstein, J. I. *et al.* A Contemporary Prostate Cancer Grading System: A Validated Alternative to the Gleason Score. *Eur. Urol.* **69,** 428–435 (2016).
3. Szegedy, C., Vanhoucke, V., Ioffe, S., Shlens, J. & Wojna, Z. Rethinking the Inception Architecture for Computer Vision. in *2016 IEEE Conference on Computer Vision and Pattern Recognition (CVPR)* (2016). doi:10.1109/cvpr.2016.308
4. Liu, Y. *et al.* Detecting Cancer Metastases on Gigapixel Pathology Images. *arXiv [cs.CV]* (2017).
5. Martin Abadi and Paul Barham and Jianmin Chen and Zhifeng Chen and Andy Davis and Jeffrey Dean and Matthieu Devin and Sanjay Ghemawat and Geoffrey Irving and Michael Isard and Manjunath Kudlur and Josh Levenberg and Rajat Monga and Sherry Moore and Derek G. Murray and Benoit Steiner and Paul Tucker and Vijay Vasudevan and Pete Warden and Martin Wicke and Yuan Yu and Xiaoqiang Zheng. TensorFlow: A System for Large-Scale Machine Learning. in (USENIX Association).
6. Geoffrey Hinton Nitsh Srivastava. Neural Networks for Machine Learning. *University of Toronto Computer Science* Available at: http://www.cs.toronto.edu/~tijmen/csc321/slides/lecture_slides_lec6.pdf. (Accessed: 14th August 2018)
7. Daniel Golovin, Benjamin Solnik, Subhodeep Moitra, Greg Kochanski, John Karro, D. Sculley. Google vizier: A service for black-box optimization. in *Proceedings of the 23rd ACM SIGKDD International Conference on Knowledge Discovery and Data Mining* 1487–1495 (Google, August 13 - 17, 2017).
8. Wang, D., Khosla, A., Gargeya, R., Irshad, H. & Beck, A. H. Deep Learning for Identifying Metastatic Breast Cancer. *arXiv [q-bio.QM]* (2016).
9. Ehteshami Bejnordi, B. *et al.* Diagnostic Assessment of Deep Learning Algorithms for Detection of

Lymph Node Metastases in Women With Breast Cancer. *JAMA* **318,** 2199–2210 (2017).

10. Ehteshami Bejnordi, B. *et al.* Deep learning-based assessment of tumor-associated stroma for diagnosing breast cancer in histopathology images. in *2017 IEEE 14th International Symposium on Biomedical Imaging (ISBI 2017)* (2017). doi:10.1109/isbi.2017.7950668
11. Chihara, L. M. & Hesterberg, T. C. *Mathematical Statistics with Resampling and R*. (2018).
